# Antiaromatic Molecules as Magnetic Couplers: A Computational Quest

**DOI:** 10.1021/acs.jpca.3c05784

**Published:** 2024-01-24

**Authors:** Suranjan Shil, Debojit Bhattacharya, Anirban Misra, Laimutis Bytautas

**Affiliations:** † Manipal Centre for Natural Sciences (Centre of Excellence), Manipal Academy of Higher Education, Manipal 576104, India; ‡ Kabi Sukanta High School, Siliguri 734010, India; § Department of Chemistry, University of North Bengal, Raja Rammohunpur, Siliguri 734013, India; ∥ Department of Chemistry, Galveston College, 4015 Avenue Q, Galveston, Texas 77550, United States

## Abstract

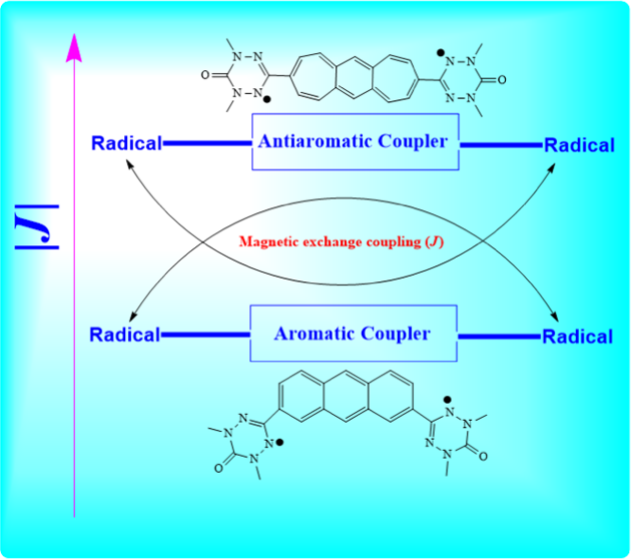

In this study, we investigate a set of organic diradical structures in which two oxo-verdazyl radicals are selected as radical spin centers that are connected (coupled) via six coupler molecules (CM), resulting in various magnetic (ferromagnetic (FM) or antiferromagnetic (AFM)) characteristics, as reflected by their exchange coupling constants (*J*). We have designed 12 diradicals with 6-antiaromatic couplers coupled with bis-oxo-verdazyl diradicals with meta–meta (m–m) and para–meta (p–m) positional connectivities. The nature of the magnetic coupling (ferromagnetic, nonmagnetic, or antiferromagnetic) and the magnitude of the exchange constant *J* depend on the type of coupler, the connecting point between each radical center and CM, the degree of aromaticity of the coupler, and the length of the through-bond distance between radical centers. The computed magnetic exchange coupling constants *J* for these diradicals at the B3LYP/6-311++G(d,p) and MN12SX/6-311++G(d,p) levels of theory are large for many of these structures, indicating strong ferromagnetic coupling (with positive *J* values). In some cases, magnetic couplings are observed with *J* > 1000 cm^–1^ (B3LYP/6-311++G(d,p)) and strong antiferromagnetic coupling (with negative *J* values) with *J* < −1000 cm^–1^ (B3LYP/6-311++G(d,p)). Similarly, in some cases, magnetic couplings are observed with *J* > 289 cm^–1^ (MN12SX/6-311++G(d,p)) and strong antiferromagnetic coupling (with negative *J* values) with *J* < −568 cm^–1^ (MN12SX/6-311++G(d,p)). Furthermore, while numerous studies have reported that the degree of aromaticity of molecular couplers often favors strong ferromagnetic coupling, displaying the high-spin character of diradicals in their ground states, the couplers chosen in this study are characterized as antiaromatic or nonaromatic. The current investigation provides evidence that, remarkably, antiaromatic couplers are able to enhance stability by favoring electronic diradical structures with very strong ferromagnetic coupling when the length of the through-bond distance and connectivity pattern between radical centers are selected in such a way that the FM coupling is optimized. The findings in this study offer new strategies in the design of novel organic materials with interesting magnetic properties for practical applications.

## 1 Introduction

The quest for designing and understanding novel organic compounds that can be characterized as open-shell singlet or triplet electronic spin states exhibiting unique properties that can be applied for various practical applications (spintronics, quantum information science, organic electronics) has long been of considerable interest to researchers.^1−32^ Some of the most important characteristics describing organic compounds include their stability, optical and magnetic properties, and degree of reactivity.^1,2^ Thus, many theoretical and computational models for predicting such properties have been developed over many years for the purpose of accurately describing the electronic structures of such systems. In this context, some of the most useful concepts for understanding organic systems are aromaticity, the degree of diradical character, and magnetic properties.^1−33^ To this end, various descriptors for evaluating the degree of aromaticity have been developed,^34−51^ as well as various measures of diradical character.^1,33^ In addition to the development of theoretical and computational methodology, encouragingly, a number of experimental techniques, like scanning tunneling microscopy, have been successfully developed and used for investigating single-molecule-magnet and single-molecule-device architectures (e.g., spin valves and spin transistors) and illuminate quantum properties of single-molecule magnets at the single-molecule level.^28^

Early attempts to understand the unusually high stability of benzene discovered by Faraday^52^ in 1825, as well as many other π-conjugated molecules, have inspired many fruitful developments (one of the earliest successful benzene models was proposed by Kekulé^53^) in chemistry, resulting in the introduction of the concept of aromaticity^54,55^ to describe the unusually high stability of some organic molecules as compared to other systems of comparable size. In 1931, Hückel introduced his famous rule^56^ to predict the stability of planar, cyclic, and π-conjugated molecules with (4*n* + 2) π-electrons. Interestingly, Hückel’s rule was recently generalized and derived^57^ from the asymmetry requirement of fermionic wave functions. Hückel’s rule has also been generalized in terms of Clar’s “aromatic sextet”.^58^ Shortly after, Breslow and co-workers^47−49^ introduced a new term called *antiaromaticity* that describes the reduced stability of some molecules as compared to the aromatic ones. In contrast to aromatic systems, antiaromatic molecules correspond to (4*n*) π-electrons in planar, cyclic, and conjugated organic systems. Many studies were dedicated to elucidating the usefulness of the concepts of aromaticity and antiaromaticity in understanding organic compounds, especially with respect to their stability and reactivity.^50,59^ In this context, we note that while it is highly desirable to order organic molecules with respect to the degree of their aromaticity in a simple “linear” order, recent studies,^60−64^ however, favor the idea that aromaticity is most accurately described as a partially ordered set of a multidimensional nature.

A significant number of recent research studies^2,3,5,8−26,29−32^ have been dedicated to finding new strategies for designing new organic diradicals with unique magnetic properties, especially in chemical processes that change or modify the magnetic properties of systems when transitions from ferromagnetic (FM) to antiferromagnetic (AFM) coupling (or vice versa) or when significant changes in the magnitude of the magnetic coupling constant *J* are observed. For example, Malik and Bu^30^ have shown that intramolecular proton transfer is able to modulate the magnetic spin-coupling interaction in photochromic azobenzene derivatives with *ortho*-site hydroxyl as a modulator, where some molecules can undergo magnetic conversion between antiferromagnetic and ferromagnetic coupling due to proton-transfer processes exhibiting large changes in *J* values. Similarly, Zhang et al.^16^ achieved redox-reaction-modulated magnetic transformations between AFM and FM magnetic coupling, noting that redox-induced structural change of a coupler leads to a change of its degree of aromaticity. In another study, Khurana et al.^23^ observed a transition from a zwitterionic ground state to a diradical antiferromagnetic state and then to a ferromagnetic state by gradually increasing the length of the coupler between two radical moieties. In another recent study, Kodama et al.^32^ described a novel diradicaloid in which two phenalenyl-radical sites were coupled antiferromagnetically by through-space coupling interactions due to the close proximity of these radical centers. A different strategy for modifying the magnetic properties of diradicaloids is to incorporate heteroatoms into organic systems, as recently demonstrated by Shil et al.^22^ and Guo et al.^65^

In this study, we pursue a strategy of theoretically designing and characterizing organic diradical systems with strong magnetic properties when using antiaromatic couplers instead of the more frequently used aromatic coupler systems to mediate the magnetic exchange coupling process between two radical spin centers. Although, in general, aromatic molecules are more often selected^8−12^ as coupler systems for constructing diradical molecules with interesting magnetic properties due to their high stability as mentioned above, in fact, some antiaromatic systems are known to be stable at room temperature^66−69^ and can be used as potential coupler systems. Furthermore, a recent study by Wang et al.^24^ suggested that “antiaromaticity could promote the diradical character of hydrocarbons, thus providing design principles not only for spintronics applications but also, more generally, for organic photovoltaics, optoelectronic material and chemical reactivity, in which organic diradicals are finding increasing relevance.” A recent article by Miao et al.^3^ noted that symmetric paramagnetic π-conjugated neutral diradicals (that are based on quinonoid molecules) can produce neutral diradicals with low-energy lying triplet states due to the gain in aromaticity of the coupler (bridge) rings by electron-pair splitting effect. Furthermore, asymmetric diamagnetic π-conjugated zwitterions made of pro-aromatic couplers (bridges) substituted by donor–acceptor groups have been developed,^2,3^ primarily for electrical/optical applications (nonlinear optics). Zeng et al.^2^ noted that the antiaromatic π-system tends to reduce antiaromaticity by modifying/changing its electronic or conformational/geometric structures and becomes aromatic with unpaired electrons within the planar configuration or nonaromatic with the distorted molecular framework. If a nonaromatic π-conjugated molecule becomes aromatic as a diradical or a zwitterion, then such a system is termed “pro-aromatic”.^2^

**Figure 1:**
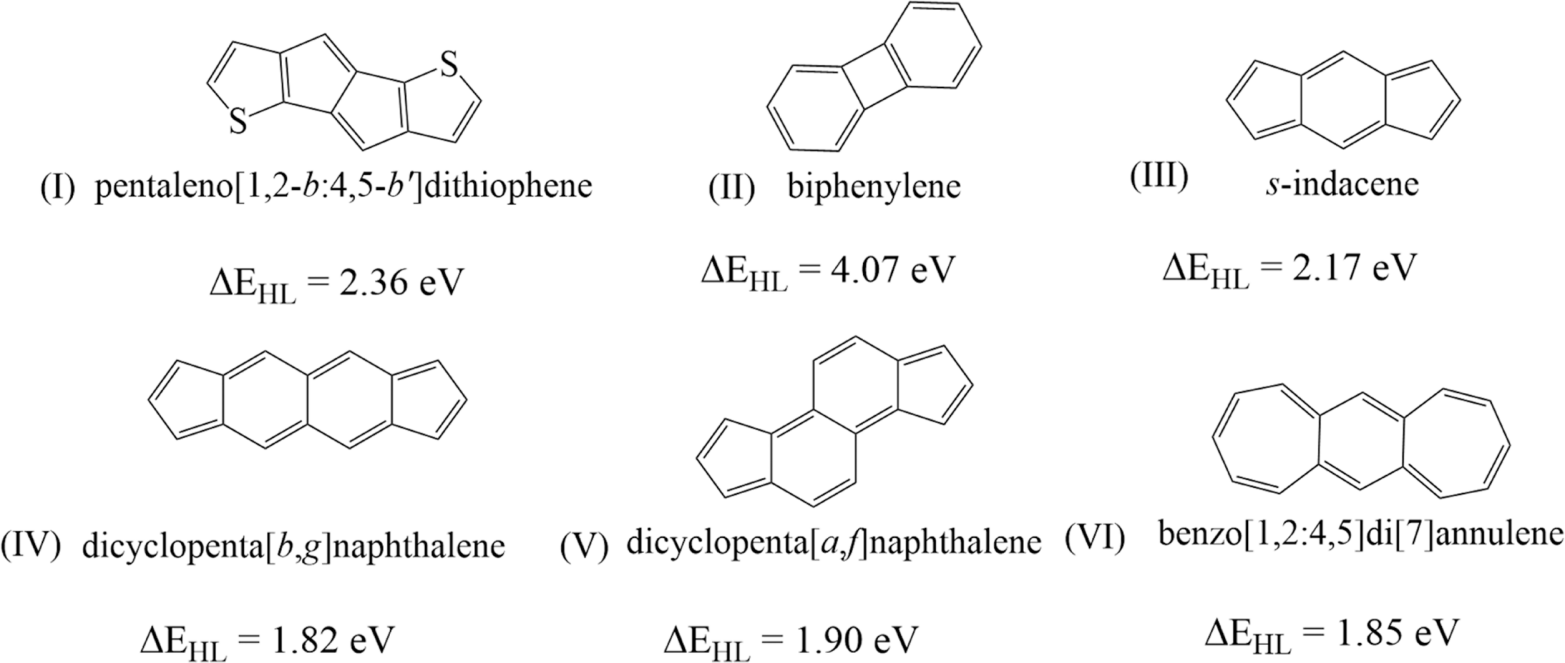
Antiaromatic couplers selected for the present study with HOMO–LUMO gaps (Δ*E*_HL_) calculated at the B3LYP/6-311++G(d,p) level are listed below each structure.

Thus, it is reasonable to argue that a highly promising strategy is to use antiaromatic couplers that, in principle, could lead to novel diradical structures with very strong magnetic (ferromagnetic or antiferromagnetic) exchange coupling between radical centers. Indeed, the exploration of novel strongly ferromagnetically or antiferromagnetically coupled diradical molecules is our main objective in this study. We have chosen six different antiaromatic couplers (see Figure 1) to design the diradicals with bis-oxo-verdazyl as the diradical spin center. It is reasonable to argue that antiaromatic coupler molecules may achieve energy (aromatic) stabilization in the system (oxo-verdazyl-radical/coupler/oxo-verdazyl-radical), where the radical sites couple either ferromagnetically or antiferromagnetically.

**Figure 2:**
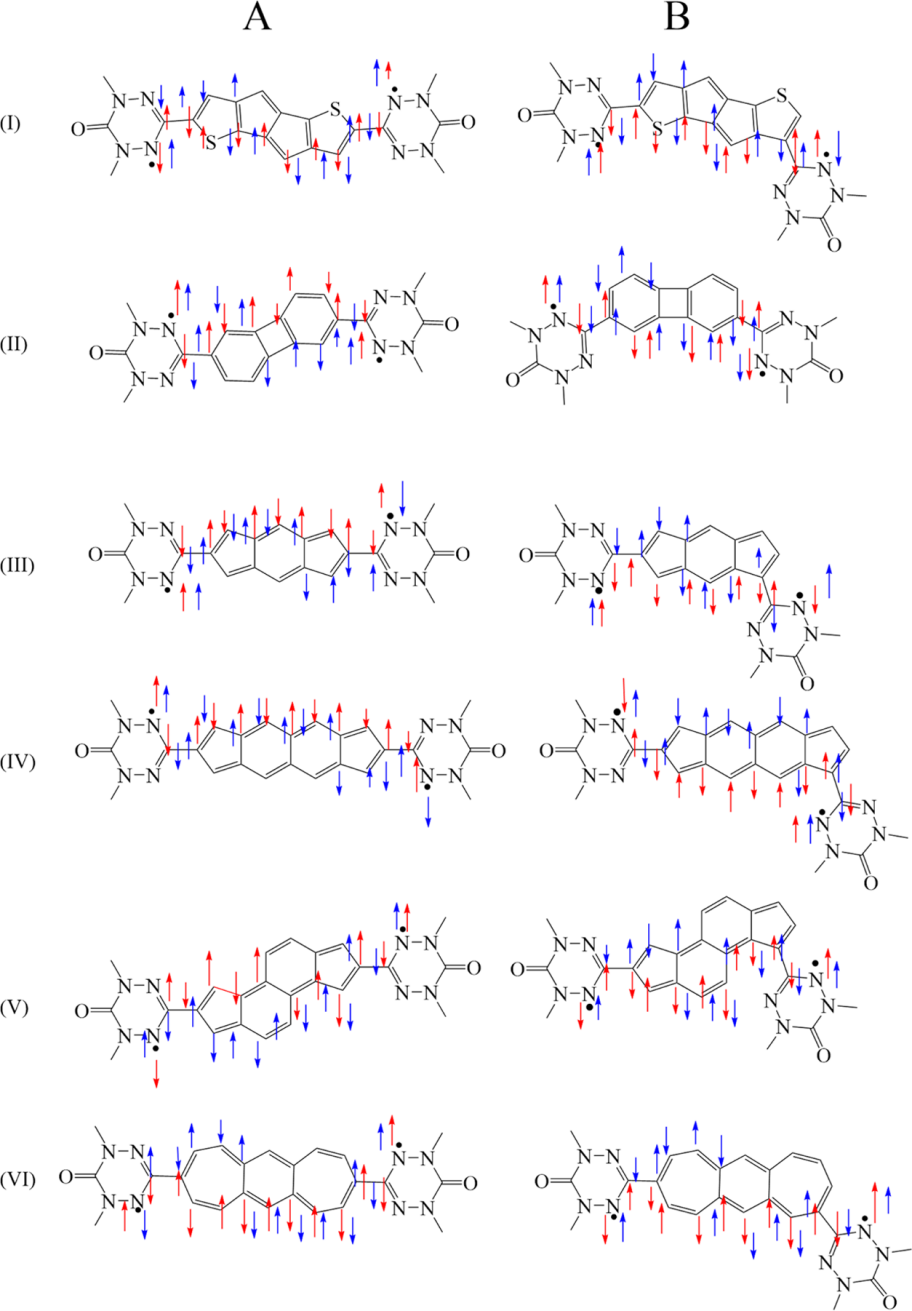
Spin polarization path for the diradicals considered in this study. A-type diradicals are shown in the left panel, and B-type diradicals are shown in the right panel. The coupling paths for the diradicals are represented in blue and red colors, where blue denotes the long spin-coupling pathway, and red denotes the short pathway.

Each of the couplers is attached to two different positions of the two oxo-verdazyl radical moieties. A total of 12 different diradical systems are considered in this study (Figure 2). The radical centers are linked through six different antiaromatic couplers (**I**–**VI**). When diradicals are constructed by accomplishing a longer through-bond shortest distance between these radical centers, as shown in Figure 2, we will label such diradicals as **I-A** to **VI-A**. On the other hand, when radical centers are being interconnected via couplers in such a way as to accomplish the shortest through-bond distance pathway between the radical centers in the construction of diradicals, as displayed in Figure 2, then we label such diradicals as **I-B** to **VI-B**. The antiaromatic couplers with 12 π-electrons are present in diradicals **I-A** and **I-B**, **II-A** and **II-B**, and **III-A** and **III-B**. On the other hand, the antiaromatic couplers with 16 π-electrons are present in diradicals **IV-A** and **IV-B**, **V-A** and **V-B**, and **VI-A** and **VI-B**.

## 2 Coupler Systems

Coupler **(I)** is a thiophene-fused pentalene^70^ stable antiaromatic compound, and the calculated HOMO–LUMO gap of the complex is 2.36 eV. Coupler **(II)** is a biphenylene, which is an interesting molecule because of its borderline character between the aromatic and antiaromatic nature with a closed-shell ground state.^71^ The calculated HOMO–LUMO gap of the compound is 4.07 eV. Coupler **(III)** is s-indacene, which is an antiaromatic compound with 12π electrons. The compound s-indacene is highly reactive and unstable in solution. The calculated HOMO–LUMO gap is 2.17 eV. Couplers **(IV)** and **(V)** have an open-shell singlet ground state.^67^ The HOMO–LUMO gaps of compounds **(IV)** and **(V)** are 1.82 and 1.90 eV, respectively. Coupler **(VI)**(72) has 16 π-electrons with a calculated HOMO–LUMO gap of 1.85 eV.

## 3 Theoretical Background

The magnetic exchange interaction between magnetic sites 1 and 2 is typically expressed using the Heisenberg–Dirac–van Vleck spin Hamiltonian, as follows
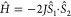
1Here, *J* represents the exchange coupling constant between the magnetic centers (1 and 2) of a diradical, and *Ŝ*_1_ and *Ŝ*_2_ are the respective spin angular momentum operators. The square of the total spin operator *Ŝ*_2_ has an eigenvalue of *S*(*S* + 1) in units of *ℏ*^2^. A positive *J* indicates ferromagnetic coupling, signifying a preference for parallel spins, while a negative value suggests antiferromagnetic interaction (just the other way around), favoring antiparallel spins. For a diradical with a single unpaired electron on each radical site, *J* can be expressed as
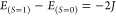
2The singlet state of a diradical cannot be accurately represented by a single Slater determinant wave function in an unrestricted formalism, leading to spin contamination. As multiconfigurational methods are computationally intensive,^73,74^ they are not employed in this study. However, Noodleman’s broken-symmetry (BS) formalism^75^ within the DFT framework provides a less computationally demanding alternative. This formalism is utilized in our study to calculate magnetic exchange coupling constants *J.*

The electronic broken-symmetry (BS) state represents the weighted average of a singlet and a triplet state, making it a non-eigenstate of the Hamiltonian. Typically, the BS solution is considered to be spin-contaminated, prompting the use of a spin-projection technique to determine the magnetic exchange coupling constant for diradicals. Various researchers have proposed different expressions for *J* based on unrestricted spin-polarized BS solutions for the lower-spin state, which also depend on the degree of overlap between magnetic orbitals (as indicated in refs (75−85)).

The works of Ginsberg,^85^ Noodleman et al.,^75^ and Noodleman and Davidson^83^ have provided expressions for *J* that are particularly valuable in cases where the overlap between magnetic orbitals is minimal. In contrast, expressions put forth by Bencini and co-workers,^82^ Ruiz et al.,^76^ supported by Illas and co-workers,^80^ are applicable when the overlap is considerable. Meanwhile, the proposition by Yamaguchi and co-workers^78,79^ is suitable for intermediate cases, bridging the gap between minimal and significant orbital overlap. In this study, we used the expression introduced by Yamaguchi and co-workers to calculate the magnetic exchange coupling constant *J*:
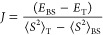
3Here, *E*_BS_ and *E*_T_, respectively, denote the total energies of the broken-symmetry (BS) singlet and nominal triplet electronic states, while ⟨*S*^2^⟩_T_ and ⟨*S*^2^⟩_BS_ represent average spin-square values for triplet and BS states, respectively.

## 4 Computational Details

The molecular geometries were optimized, and relevant physical properties were calculated using two DFT functionals, B3LYP^86,87^ and MN12SX^88^, with unrestricted formalism. The 6-311++G(d,p) basis set^89,90^ was used for the DFT calculations. NICS (nuclear independent chemical shift) values that describe magnetic indices representing a degree of aromaticity were calculated using the gauge independent atomic orbital (GIAO) methodology.^91^

The NICS value denotes negatively signed absolute shielding, which is observed at a very specific location within a given molecular system. A greater degree of aromaticity is indicated by a more negative (less positive) NICS value, reflecting the increased strength of the ring current associated with π electrons in the rings of aromatic compounds. The NICS values were calculated for each ring present in a coupler system (with and without radical moieties interconnected via a coupler).

The notation NICS(+1) denotes that the measurement was taken with the probe positioned 1 Å above the center of the ring. The rationale for calculating the NICS index at this distance lies in the magnified impact of the ring current due to the π-electrons. Additionally, at a distance of 1 Å, the influence of σ aromaticity can be effectively disregarded. Furthermore, the zz-component of NICS, denoted as NICSzz (specifically NICSzz(+1)), was also computed. The value of NICSzz(+1) represents the zz-component of the NICS tensor, calculated at a distance of 1 Å above the center of the ring.

We computed the harmonic oscillator model of aromaticity (HOMA) index alongside NICS to ensure the consistency of results. The HOMA values are determined using the following formula.^92^
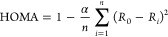
4where α and *R* are constants specific to C–C bonds in a hydrocarbon π system, *n* is the number of π bonds, *R_i_* is the observed or calculated length of the *i*th C–C bond in a given ring, and summation is made over all of the π bonds. A greater positive HOMA value indicates close to one higher local aromaticity of the ring involved. We adopted the values α = 257.7, *R*_0_(C–C) = 1.388 Å, and *R*_0_(C–S) = 1.677 Å to calculate HOMA indices.^93^ We used Multiwfn3.7^94^ to calculate HOMA at the B3LYP/6-311++G(d,p) optimized geometry.

## 5 Results and Discussion

### 5.1 Magnetic Exchange Coupling Constants

Electronic structure theory based on DFT functionals has been used extensively for calculating the magnetic properties of organic diradicals. The exchange coupling coefficient (spin-coupling constant) (*J)* is one of the most important and frequently used quantities for describing the magnetic properties of diradical molecular systems. A positive *J* value indicates that the triplet ground state is energetically below the lowest singlet state. A negative *J* value signifies that the singlet state is in the ground electronic state.

In this study, we considered a total of 12 diradicals (**I-A** to **VI-A** and **I-B** to **VI-B**) using 6 different antiaromatic couplers. The magnetic exchange coupling constant (*J*) values were calculated using two methodologies, namely, B3LYP/6-311++G(d,p) and MN12SX/6-311++G(d,p), within the unrestricted DFT formalism. The results of these calculations are displayed in Tables 1 and S1–S4. These tables list the total energy values (in hartree) for triplet electronic states and broken-symmetry (BS) states; in addition, they provide their corresponding ⟨*Ŝ*_2_⟩ values to compare with “pure-spin” state values. For instance, for pure-singlet states, we have ⟨*Ŝ*_2_⟩ = 0, and for pure-triplet states, we have ⟨*Ŝ*_2_⟩ = 2. These values provide guidance for verifying the validity of the applicability conditions for the Yamaguchi formula. In particular, Yamaguchi’s formula conditions assume that the expected ⟨*Ŝ*_2_⟩ value for the nominal triplet should be close to 2 (the expected ⟨*Ŝ*_2_⟩ value of the UHF triplet states should be close to 2). In contrast, the UHF broken-symmetry (BS) states should have expectation ⟨*Ŝ*_2_⟩ values that are close to 1, that is, halfway between a singlet and a triplet. Tables S1–S4 contain *J* values calculated using Yamaguchi’s formula in eq 3. These tables show that, for unrestricted B3LYP functional, the results for ⟨*Ŝ*_2_⟩ values (on average) display larger deviations from these values as compared to those of the restricted MN12SX functional. The ⟨*Ŝ*_2_⟩ values of diradical **IV**–**VI** show spin contamination, which is due to the radical character of the couplers.^13,67^ Nevertheless, the comparison based on the data from Table 1 (B3LYP/6-311++G(d,p) and (MN12SX/6-311++G(d,p))) indicates that these *J* values follow the *same* trends as calculated using both of these functionals. As far as the functionals discussed above are concerned, it is very difficult to determine which of them (B3LYP or MN12SX) produces more accurate results. Different results can be obtained by different functionals due to the difference in the percentage of local and nonlocal parts of the exchange and correlation contributions included in the formulation of these functionals (for more details, see the discussion in ref (22)). The other properties of these diradicals were calculated *solely* using the unrestricted B3LYP functional. Since the selected 12 diradicals are planar and each structure has a π-conjugated coupler between radical sites, it is reasonable that the *spin alternation* rule given by Trindle and Datta and co-workers^95,96^ could possibly be used to predict the nature of the magnetic exchange coupling between radical centers in diradicals, at least in some of the cases considered in this study. The effect of heteroatoms along a spin-alternation path when more than one ECP is available was investigated in ref (22) however, here we are dealing with antiaromatic spacers bearing diradicals that have more than one homoatomic spin-alternating path between radical sites.

**Table 1:** Changes in the Nuclear Independent Chemical Shift ΔNICS_*zz*_(1) (values in ppm) with Respect to Pure Couplers and Intramolecular Magnetic Exchange Coupling Constants (*J*, cm^–1^)^a^

coupler/diradical		ΔNICSzz(1), ppm	ΔNICSzz(1), ppm average	*J*, cm^–1^ UB3LYP/6-311++G(d,p)	*J*, cm^–1^ UMN12SX/6-311++G(d,p)
I	**I-A**	(+3.85, −3.41, −3.41, +3.85)	+0.22	9.72	0.00
	**I-B**	(+3.89, −2.87, −2.87, +1.80)	–0.01	–10.76	–4.56
II	**II-A**	(+1.54, −3.09, +1.54)	0.00	21.79	18.00
	**II-B**	(+1.53, −2.98, +1.53)	+0.03	–26.72	–20.80
III	**III-A**	(−5.25, −4.45, −5.25)	–4.98	–95.8	–41.69
	**III-B**	(−9.93, −15.98, −15.82)	–13.91	1200.77	153.38
IV	**IV-A**	(−0.07, −0.16, −0.16, −0.07)	–0.12	–923.02	–218.08
	**IV-B**	(−8.05, −14.65, −16.37, −9.56)	–12.16	350.29	272.24
V	**V-A**	(−3.92, −1.52, −1.52, −3.92)	–2.72	15.09	54.08
	**V-B**	(−0.28, −17.72, −18.63, −9.62)	–11.56	218.75	193.02
VI	**VI-A**	(−21.49, −17.94, −21.72)	–20.38	–1181.17	–568.12
	**VI-B**	(−30.38, −26.28, −29.95)	–28.87	571.42	289.18

a Optimized geometries and *J* values are calculated at the UB3LYP/6-311++G(d,p) Level of Theory.

Looking at the diradical structures displayed in Figure 2, we notice that there are many different types of pathways with respect to spin alternation. In accordance with the spin-alternation rule, if an even number of atoms exist between two radical centers along a spin-alteration pathway (SAP) or exchange coupling pathway (ECP), then the spin centers will couple antiferromagnetically. On the other hand, if an odd number of atoms exist between two radical centers along a spin-alteration pathway, then the spin centers will couple ferromagnetically. Examination of the diradical structures displayed in Figure 2 reveals that for each diradical structure, there are numerous ECP pathways. For example, for diradical **II-A**, there are several different ECPs, each connecting *n*-atoms (including the radical sites) along the ECP. Two such different ECPs (in red and blue), each connecting 11-atoms (including the radical sites) along the ECPs, are displayed in Figure 2. However, for the **II-A** structure, there are many more ECPs than those shown in Figure 2. For example, for the **II-A** diradical, there are also different ECPs that connect 13-atoms (including the radical sites) along the ECPs that are not shown in the figure. The odd number of atoms along the ECPs implies that ferromagnetic coupling would be favored in the **II-A** diradical. Indeed, this prediction is confirmed by the *J* values listed in Table 1. For diradical **II-B**, there are also several different ECPs. One can find ECPs that connect 10-, 12-, and 14-atoms (including the radical sites) along the ECPs and two of these spin-alternation pathways (in blue and red) for diradical **II-B** are displayed in Figure 2. The even number of atoms along the ECPs implies that antiferromagnetic coupling is expected in the ground state of the **II-B** diradical. Again, as expected, this prediction is confirmed by the *J* values listed in Table 1. Thus, the spin alternation rule is helpful in predicting that diradical **II-A** should exhibit ferromagnetic character and that diradical **II-B** should display antiferromagnetic interactions between radical centers.

Now, let us consider the **I-A** diradical displayed in Figure 2. Here, the situation for using the spin-alternation rule is no longer simple. One can find ECPs that connect 12-, 13-, 14-, and 16-atoms (including the radical sites) along the different ECPs. Two of these spin-alternation pathways (in blue and red) connecting 12- and 13-atoms, for diradical I-A, are displayed in Figure 2. Thus, there is no simple “back-of-the-envelope” method to predict the nature of magnetic exchange coupling in diradical **I-A**. For the **I-B** diradical displayed in Figure 2, the data in Table 1 indicate the *J* values with antiferromagnetic coupling. For the **I-B** structure, one can find ECPs that connect 11-, 12-, 13-, 14-, 16-, and 17-atoms (including the radical sites) along different ECPs. Figure 2 displays only two different ECPs connecting 11- and 12-atoms along different pathways. Thus, there is no simple rule to predict the nature of magnetic exchange coupling in diradical **I-B** based on a spin-alternating-pathway rule due to the existence of many *odd* and *even* number of atoms along different ECPs, as is the case for diradical **I-A**.

Analyzing the *J* values for diradicals **III-A** and **III-B**, one observes that the spin coupling in **III-A** is antiferromagnetic, while the spin coupling in **III-B** is ferromagnetic. Looking at the different spin-alternation (ECP) pathways for the **III-A** structure, one can find ECPs that connect 11-, 12-, and 13-atoms (including the radical sites) along different ECPs. Similarly, for the **III-B** diradical, one can find ECPs that connect 8-, 9-, 10-, 11-, and 13-atoms (including the radical sites) along different ECPs. Thus, the simple spin-alternating-pathway rule cannot be used to predict the nature of magnetic exchange coupling in diradicals **III-A** and **III-B**.

Based on the calculated *J* values for diradicals **IV-A** and **IV-B**, one observes that the spin coupling in **IV-A** is *antiferromagnetic*, while the spin coupling in **IV–B** is ferromagnetic. Looking at the different spin-alternative (ECP) pathways for the **IV-A** structure, one can find ECPs that connect 13-, 14-, 15-, and 16-atoms (including the radical sites) along different ECPs (see Figure 2 for ECPs containing 13- and 14-atoms along their pathway). For the **IV-B** diradical, one can find ECPs that connect 12-, 13-, 15-, 16-, and 17-atoms (including the radical sites) along different ECPs (see Figure 2 for ECPs containing 13- and 14-atoms along their pathway). Thus, the simple spin-alternating-pathway rule cannot be used to predict the nature of magnetic exchange coupling in diradicals **IV-A** and **IV-B**.

A similar situation also exists for diradicals **V-A** and **V-B** as well as diradicals **VI-A** and **VI-B;** namely, the simple spin-alternating-pathway rule cannot be used in a straightforward way to predict the nature of magnetic exchange coupling in these diradicals (see Figure 2 for a few selected pathways shown in red and blue). In conclusion, the simple spin-alternating-pathway rule can be used to predict the nature of magnetic exchange coupling in diradicals **II-A** and **II-B**. In order to illustrate the concept of the spin-alternating-path rule, Table 2 displays only the number of coupler atoms for a few of the ECPs discussed above (here, the number of atoms along the ECPs that is listed are reduced by 4 compared to all atoms along a spin-alternating pathway since only coupler atoms are counted). The diradicals **V-A** and **V-B** result in a similar sign of magnetic exchange as the length of the coupling path (see Figure 2) remains unaltered.

**Table 2:** Illustration of the Concept of the Spin-Alternation-Path Rule for Selected ECP Cases: Correlation of the Number of Coupler’s Atoms along the Selected ECPs between Radical Centers and the Nature of Magnetic Coupling^a^

		series A		series B	
diradicals	coupling route	coupler atoms	magnetic coupling	coupler atoms	magnetic coupling
I	red	8	ferromagnetic	7	antiferromagnetic
	blue	9		8	
II	red	7	ferromagnetic	6	antiferromagnetic
	blue	7		8	
III	red	7	antiferromagnetic	6	ferromagnetic
	blue	8		7	
IV	red	9	antiferromagnetic	8	ferromagnetic
	blue	10		9	
V	red	8	ferromagnetic	8	ferromagnetic
	blue	9		9	
VI	red	9	antiferromagnetic	8	ferromagnetic
	blue	10		9	

a Here, the number of atoms along the ECPs listed is reduced by 4 compared to all atoms along a spin-alternating pathway since only coupler atoms are counted.

As can be seen from the data listed in Table 1, diradicals **I-A/I-B** and **II-A/II-B** exhibit very small absolute values for the exchange coupling constant *J*. On the other hand, the diradicals **III-A/III-B**, **IV-A/IV-B**, **V-A/V-B**, and **VI-A/VI-B** clearly display significantly larger magnitudes of *J* values for both UB3LYP and UMN12SX functionals. Notably, the B-series diradicals exhibit much stronger ferromagnetic type of exchange coupling, i.e., larger positive *J* values as compared to that of the A-series diradicals that mostly (except for the **V-A** diradical) are characterized by antiferromagnetic coupling, i.e., negative *J* values. It can be argued that factors like the radical character of the coupler and the degree of aromaticity of the coupler can play a significant role in determining the coupling constant values for the diradicals considered in this study.^8,15^ In particular, the degree of aromaticity increase observed in the couplers when constructing the B-series diradicals as compared to that of the couplers forming the A-series diradicals will be the focus of the next section.

### 5.2 Aromaticity and Magnetism

Aromaticity, magnetism, and chemical reactivity are of fundamental significance when exploring the physical and chemical properties of π-conjugated diradical systems and have been the subject of numerous studies.^97−99^ The concept of bond breaking, for example, in the ring of an organic molecule, would lead to the formation of a diradical, or possibly zwitterion, and has preoccupied chemists trying to understand the electronic structure of molecules from first principles with, for example, articles having intriguing titles like “Do diradicals behave like radicals?^1^” It is quite common to explore the breaking of chemical bonds in terms of the formation of radical fragments that require highly accurate accounting of electron correlation (both static or nondynamic as well as dynamic kind)^73,74^ when exploring potential energy surfaces in chemical systems. In this paper, however, we will focus on stability (aromaticity and antiaromaticity) and magnetic properties of molecular systems representing minima on potential energy surfaces.

In general, there has been much interest in understanding the relationship between the emergence and coupling of unpaired electrons in molecular systems and aromaticity or antiaromaticity. Recently, Zeng and co-workers^2^ noted that pro-aromatic and antiaromatic molecules exhibit an irresistible tendency to become diradicals in their ground electronic state, thus, emphasizing that the diradical character emerges as an important concept in chemistry characterizing organic opto-magnetic molecular systems. Such observation is consistent with studies^97−99^ explaining the emergence of unpaired π-electron densities on graphene edges when the competition between local pairing and delocalized resonance yields unpaired electrons at the graphene edges of sufficiently wide graphene strips, for example, unpaired electrons on so-called “zig-zag” edges within the network of alternate π-networks, where carbon sites can be divided into starred and unstarred sets, where no member of either set is found being adjacent to a member of the same set. Thus, translationally symmetric graphene strips are predicted^97^ to exhibit unpaired π-electron densities on opposite edges when the width of the strip gets sufficiently large for certain types of edge shapes, like “zig-zag” edge or “Klein edge”. Notably, in such cases, the resonance stabilization in the bulk of the graphene strip results in the *emergence of unpaired* π-electrons that can be confirmed experimentally.^100^

In the context of diradical molecular systems, there has been much interest in investigating molecular systems that result from the coupling of two individual monoradical sites using molecular couplers as mediators. Much of the research^5−25^ has focused on exploring the correlation between the diradical magnetic properties (ferromagnetism, nonmagnetism, and antiferromagnetism) and degree of aromaticity (or antiaromaticity) of the couplers (spacers) that mediate magnetic exchange coupling (represented by the value of the magnetic coupling constant *J*) between monoradical centers. An early study by Ali and Datta^8^ analyzing the influence of the length and aromaticity of coupler molecules found that π-conjugation and spin polarization play a major role in determining the magnitude and nature (ferromagnetic vs antiferromagnetic) of magnetic coupling between monoradical centers in a diradical. Using bis-nitronyl nitroxide diradicals in their study, the authors found that, in general, the aromaticity of couplers has a strong correlation with their magnetic properties. In particular, the aromaticity of couplers favors a ferromagnetic trend, while the lowering of the degree of aromaticity supports antiferromagnetic coupling between radical centers. Other studies^17,18^ have further supported these conclusions. Studies in refs (17,18) found that the correlation between the magnetic exchange coupling constant *J* and the degree of aromaticity of the couplers (spacers) indeed can be observed in many different π-conjugated diradical systems. In addition, it is interesting to note that *heteronuclear* couplers are, in general, *less aromatic* than homonuclear (carbon-based) couplers.^8^

Since the concepts of aromaticity and magnetism are of fundamental significance when discussing the properties of molecular systems, both subjects have enjoyed much attention as evidenced by many publications on the subject. For example, considerable effort^34−51^ has been dedicated to developing powerful descriptors to represent the degree of aromaticity in the most predictive, desirably in a “linear-type-correlation” fashion, where an aromaticity index is capable of ordering unique molecular structures from the least to the most aromatic. For example, von Ragué Schleyer et al.^51^ argued that energetic, geometric, and magnetic criteria yield the same ordering of five-membered ring systems C_4_H_4_X with respect to aromaticity/antiaromaticity. These authors found that for specific selected X-fragments, the aromatic 6-π electron systems are stabilized, their bond lengths are equalized, and magnetic susceptibility characteristics are *diamagnetic.* On the other hand, the antiaromatic 4-π electron systems are destabilized, double bonds are localized, and magnetic susceptibility characterization is paramagnetic. As noted earlier, indeed, several studies^8,16−18^ of diradicals confirm that there is a close relationship between the aromaticity of couplers that link the unpaired-spin radical centers and the exchange coupling coefficients *J*. Studies have shown that an *increase* in the aromaticity of such couplers favors a ferromagnetic trend, while a *decrease* in aromaticity favors antiferromagnetic tendencies. Although many aromaticity descriptors can be found in the literature, some recent studies^39,40,45^ suggest that the NICS_*zz*_ index is one of the best indicators for describing the correlation between the magnetic properties of diradicals and the aromaticity of π-conjugated couplers in diradical systems. In particular, the NICS_*zz*_(+1) component is strongly recommended^43^ as one of the most accurate indicators in reflecting the degree of aromaticity among the NICS indices. Thus, in this study, we chose NICS_*zz*_(+1) to measure the degree of aromaticity of each coupler. In particular, the more negative (or less positive) the value of NICS_*zz*_(+1), the higher the degree of aromaticity of the system.

**Figure 3:**
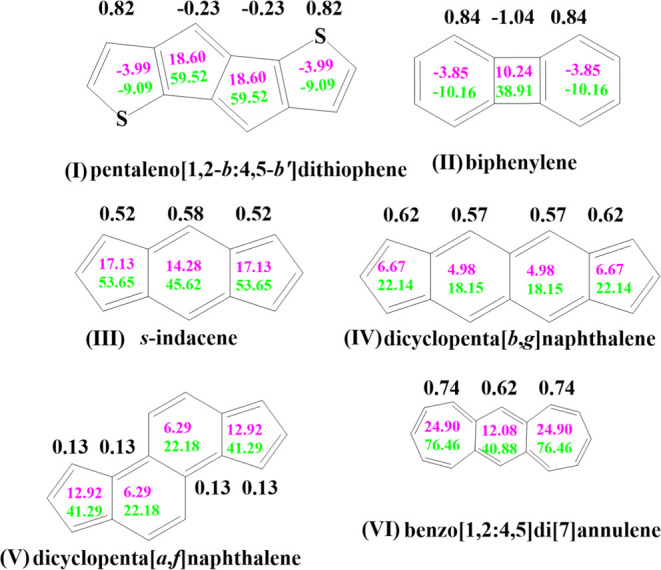
NICS and HOMA values for pure couplers. The color code is as follows: pink, NICS(1); green, NICS_*zz*_ (1); black, HOMA.

**Figure 4:**
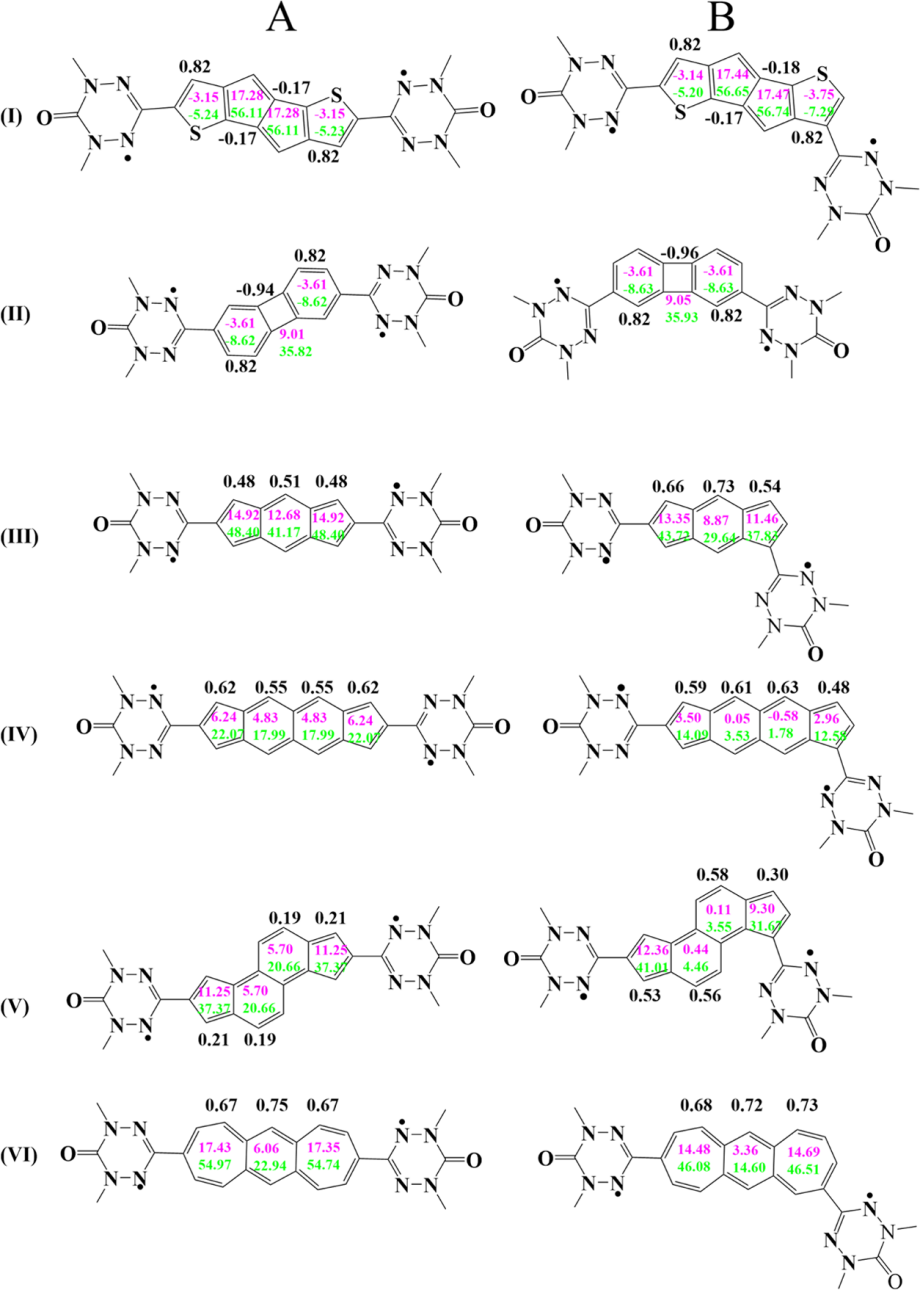
NICS and HOMA values for couplers that are attached to radical moieties. The color code is as follows: pink, NICS(1); green, NICS_*zz*_ (1); black, HOMA.

The relevant structures, as well as the NICS(1) and NICS_*zz*_(+1) data, are presented in Figures 3 and 4. Here, NICS(+1) values are in *pink* color and NICS_*zz*_(+1) values are in *green* color for each ring structure in each of the six couplers selected for the present study. Figure 3 displays only structures for pure couplers (structures **I** to **VI**, i.e., six structures in total), while Figure 4 lists the complete structures for diradicals of A-type (**I-A** to **VI-A**) and B-type (**I-B** to **VI-B**) with a total of 12 structures. Compared to the earlier studies in refs (17,18) exploring aromaticity of couplers in organic diradicals, where the couplers were mostly aromatic molecules with NICS_*zz*_(+1) values ranging from −2 to −10 ppm at the UB3LYP level of theory, in this study, we see NICS_*zz*_(+1) values for the couplers (pure couplers, couplers in **A-type** diradicals, or couplers in **B-type** diradicals) that mostly exhibit large positive values ranging from −10 to +76 ppm. Since the couplers selected in this study are indeed antiaromatic in nature, it is very satisfying that the NICS_*zz*_(+1) values clearly reflect the high degree of antiaromaticity (i.e., they have large positive values of NICS_*zz*_(+1)). As our objective is to explore the correlation of *J* values for diradicals of **A-type** (**I-A** to **VI-A**) and **B-type** (**I-B** to **VI-B**) with changes in the aromatic stability of the couplers that are used to mediate the exchange coupling between the radical sites, it is most meaningful to examine *changes* in the values of NICS_*zz*_(+1) going from pure coupler to either **A-type** diradical or to **B-type** diradical for each ring. Then, examining trends of how these changes in NICS_*zz*_(+1) values correlate with the calculated *J* values for A-type and B-type diradicals will provide important insight into this correlation. Thus, for each *coupler-C*, which is part of a diradical-K, we can define the following quantity: ΔNICS_*zz*_(coupler-C, ring-R, diradical-K). Thus, for each diradical-K containing coupler-C and for each ring-R contained in coupler-C, we define
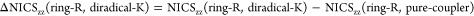
5Here, for clarity, we omitted +1 Å from the notation in NICS_zz_(+1). Also, for simplicity, we omitted the coupler-C symbol in ΔNICS_zz_(coupler-C, ring-R, diradical-K). Since each coupler has many rings, in addition to the individual values ΔNICS_zz_(ring-R, diradical-K) listed for each ring R in coupler-C, we also list averaged values NICS_zz_(+1) per ring for each coupler-C in a diradical structure (**I-A**, **II-A**, **III-A**, **IV-A**, **V-A**, **IV-A**, **I-B**, **II-B**, **III-B**, **IV-B**, **V-B**, **IV-B**) in Table 1 to simplify our discussion a bit, but without any loss of rigor.

Examining the average ΔNICS_*zz*_(1) values for diradicals **I-A**, **I-B**, **II-A**, and **II-B** in Table 1, we observed that these values are exceedingly small. Similarly, the exchange coupling constants *J* for these diradical systems have very small magnitudes. Thus, for the **I-A**, **I-B**, **II-A**, and **II-B** diradicals, there seems to be no clear correlation between changes in the aromaticity due to a coupler participating in exchange coupling and the *J* values for these diradicals.

We would like to add a comment regarding the exchange coupling *J* values for systems **I-A** and **I-B** since the coupler molecules in these diradicals contain sulfur atoms in their ring structures. The discussion in ref.^22^ already noted that the presence of heteroatoms along a spin-alternating pathway tends to lower the *J* values, labeling it a “heteroatom blocking effect”. As shown in Table 1, the magnitudes of the *J* values for diradicals **I-A** and **I-B** are indeed very low, arguably due to the presence of the sulfur atom along with some of the spin-propagation paths (there are a total of 2 sulfur atoms) compared to other diradicals considered in this study. Since sulfur is placed in the third period of the periodic table with a larger atomic size (as compared to a carbon atom), this will cause a mismatch in the size of its valence electron density as compared to that of carbon atoms. As a consequence, this mismatch in size will hinder the itinerant spin exchange interaction between spin centers, which results in zero-spin density on the sulfur atom if ECPs include S atoms. Thus, the extremely small *J* values for structures **I-A** and **I-B**, namely, 9.72 and −10.76 cm^–1^ (UB3LYP/6-311++G(d,p)) and 0.0 and −4.56 cm^–1^(MN12SX/6-311++G(d,p)), respectively, strongly support this argument.

Now, let us examine the diradical structures (**III-A** and **III**-**B**), (**IV-A** and **IV-B**), (**V-A** and **V-B**), and (**VI-A** and **VI-B**), and compare the *J* values at the UB3LYP/6-311++G(d,p) level of theory for each of these diradical pairs displayed in Table 1. For the (**III-A** and **III-B**) pair, the *average* ΔNICS_*zz*_(1) values are −4.98/–13.91 ppm, while the *J* constants are −95.8/1200.8 cm^–1^. For the **(IV-A** and **IV-B**) pair, the *average* ΔNICS_*zz*_(1) values are −0.12/–12.16 ppm, while the *J* constants are −923.0/350.3 cm^–1^. For the (**V-A** and **V-B**) pair, the *average* ΔNICS_*zz*_(1) values are −2.72/–11.56 ppm, while the *J* constants are 15.1/218.8 cm^–1^. For the (**VI-A** and **VI-B**) pair, the *average* ΔNICS_*zz*_(1) values are −20.38/–28.87 ppm, while the *J* constants are −1181.2/571.4 cm^–1^. On average, the couplers **III**, **IV**, **V**, and **VI** undergo aromatic stabilization in both types (**A-type** and **B-type**) of diradicals. However, a much more significant aromatic stabilization in terms of the average ΔNICS_*zz*_(1) values (by about −10 ppm) was observed for coupler molecules in the **B-type** partner in each (**A-type/B-type**) pair. Furthermore, the exchange coupling constants *J* for the **B-type** of the diradical partner in each (**A-type/B-type**) pair display a very strong ferromagnetic coupling with *J* values ranging from 219 to 1200 cm^–1^. While the discussion above used the *J* data calculated using the UB3LYP functional, the main conclusions remain the same for the data calculated using the UMN12SX functional.

We also calculated the HOMA (harmonic oscillator model of aromaticity, see ref (18)) aromaticity indices for the pure coupler rings and for the rings in the **A-type/B-type** diradicals. The HOMA values that are closer to 1 represent higher aromaticity levels. The calculated HOMA values for the rings in the B-series diradicals, in general, exhibit values that are closer to 1 than the A-series diradicals, thus favoring a higher degree of aromaticity estimate for the B-series diradicals. For example, the six-membered ring in the **III-B** diradical has a HOMA value of 0.73, while the six-membered ring in the **III-A** diradical has a HOMA value of 0.51 (the HOMA value for the six-membered ring in the pure coupler-**III** is 0.58). Similarly, the two six-membered rings in the **V-B** diradical have HOMA values of 0.56 and 0.58, respectively, while both the two six-membered rings for the **V-A** diradical have HOMA values of 0.19 (the HOMA values for both the six-membered rings in the pure coupler-**V** is 0.13). Although the HOMA aromaticity indices for the coupler ring in the remaining diradicals have rather small variations between the **A-type** and **B-type** diradicals, the degree-of-aromaticity estimates based on the HOMA indices are consistent with the degree-of-aromaticity trends based on the NICS_zz_(+1) values discussed above. This evidence supports the conclusion that **B-type** diradicals exhibit a higher degree of aromatic stabilization than **A-type** diradicals.

Thus, based on this evidence, we can state that a very strong ferromagnetic coupling in the **B-type** diradicals considered in this study is associated with a significant increase in the degree of aromaticity of the coupler fragment. Furthermore, it is important to emphasize that the antiferromagnetic J values display remarkably large magnitudes for diradicals that contain antiaromatic couplers, which is significant and compares very well with the strong ferromagnetism observed in similar-size systems. For example, Latif et al.^20^ reported a very strong ferromagnetic coupling for diradicals, where nitronyl nitroxide was coupled to oxo-verdazyl through polyene spacers with J values reaching up to 1157 cm^–1^. Thus, the findings in this study are very encouraging in shedding light on the development of organic diradicals with strong ferromagnetic coupling using antiaromatic couplers.

### 5.3 SOMOs and LUMOs, SOMO–SOMO, and HOMO–LUMO Energy Level Splitting

An interesting consideration in predicting ferromagnetic or antiferromagnetic exchange coupling between radical centers in a diradical is the examination of patterns and shapes of SOMOs (singly occupied molecular orbitals) as well as the spin alternation rule in the unrestricted Hartree–Fock (UHF) treatment.^95,96^

For example, a theoretical study performed by Ali and Datta^8^ of diradicals involving bis-nitronyl nitroxide diradicals (with selected couplers) found that ferromagnetic interaction is observed when the shapes of the SOMOs are of disjoint type. Such a prediction has received supporting evidence^8^ from experimental data as well as from the spin alternation rule. Here, we note that the disjoint SOMO–SOMO pair set contains orbitals with orbital coefficient contributions containing no atoms that are common, while the nondisjoint SOMO–SOMO pair set contains orbital contributions that are common with some (or all) atoms in a system. The smaller the HOMO–LUMO gap of the coupler (see Figure 1), the higher the observed coupling constant for the diradicals in this study, which is in accordance with our previous results.^15^

**Figure 5:**
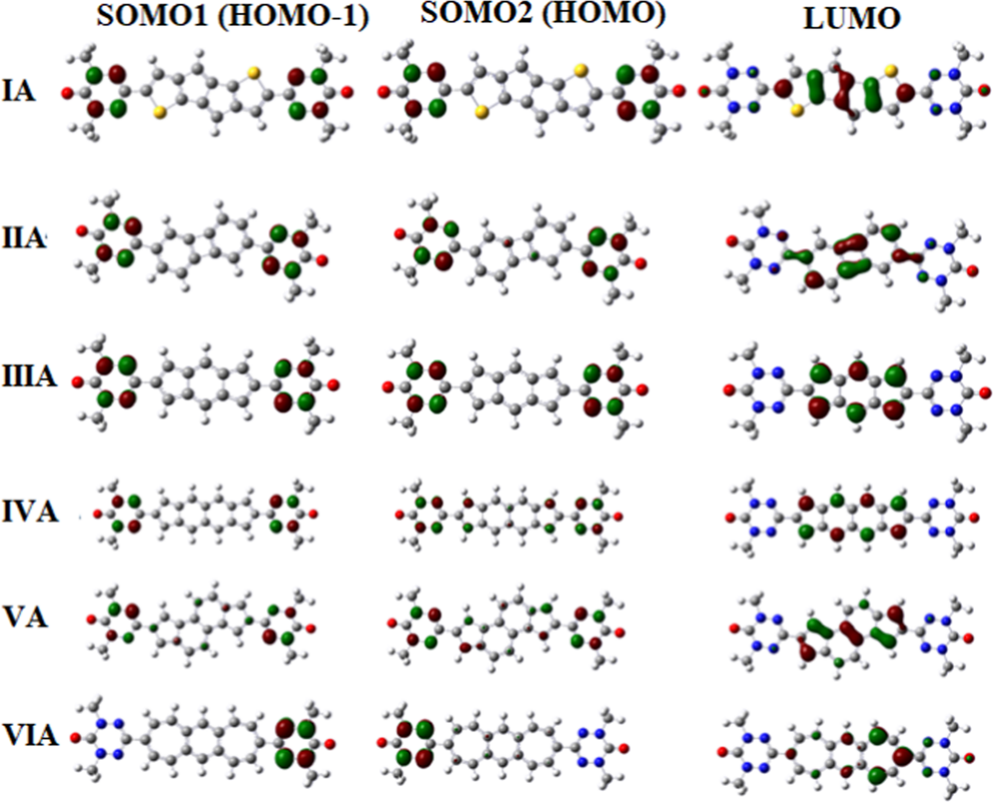
Molecular orbitals (SOMO-1, SOMO-2, and α-LUMO) for structures I-A to **VI-A** at the B3LYP/6-311++G(d,p) level of theory in the high-spin state. The iso-value for the MO plots is set at 0.05. Red, gray, blue, white, and yellow atoms represent the oxygen, carbon, nitrogen, hydrogen, and sulfur atoms, respectively.

**Figure 6:**
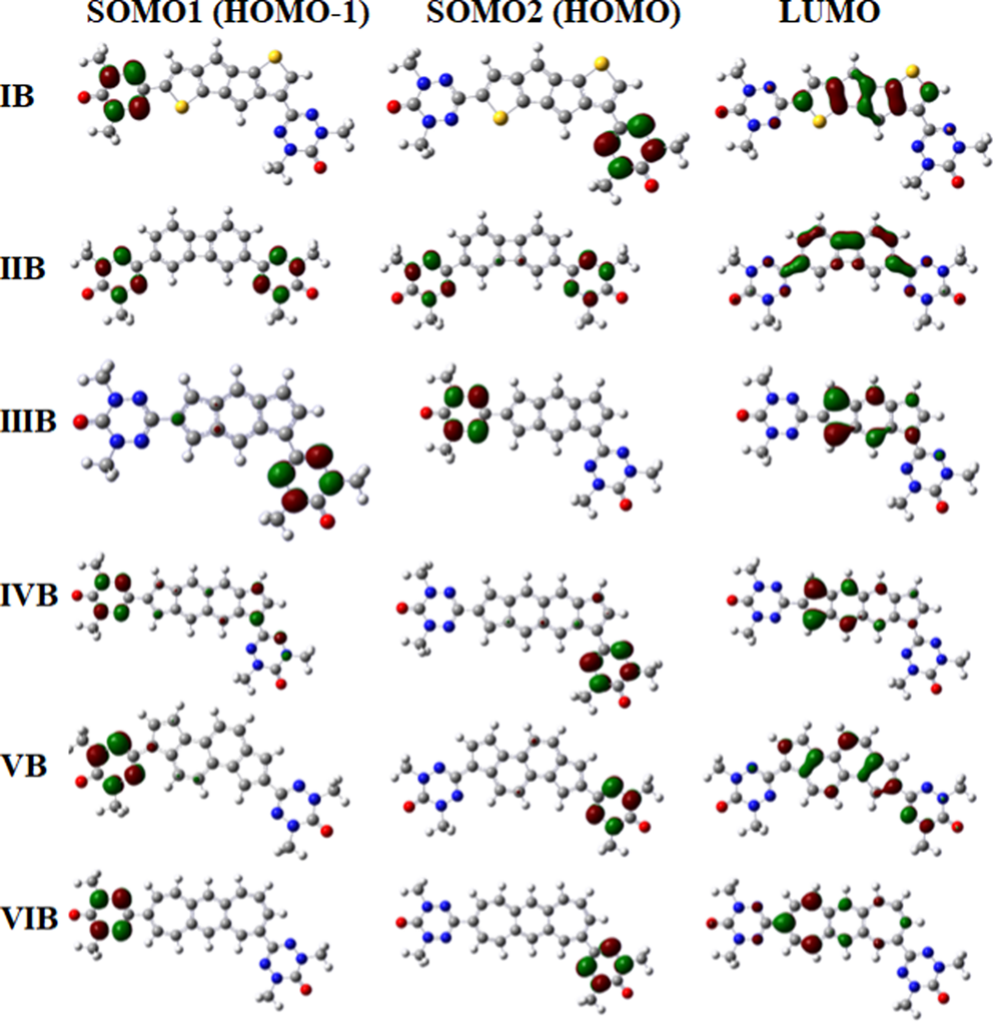
Molecular orbitals (SOMO-1, SOMO-2, α-LUMO) for structures **I-B** to **VI-B** at the B3LYP/6-311G++(d,p) level of theory in the high-spin state. The iso-value for the MO plots is set at 0.05. Red, gray, blue, white, and yellow atoms represent the oxygen, carbon, nitrogen, hydrogen, and sulfur atoms, respectively.

Figure 5 displays the molecular orbitals (SOMO-1, SOMO-2, LUMO) for diradical structures **I-A** to **VI-A**, while Figure 6 displays the molecular orbitals (SOMO-1, SOMO-2, LUMO) for structures **I-B** to **VI-B**. Also, Table 3 displays the SOMO-1, SOMO-2, and LUMO energies (in au) as well as the SOMO–SOMO and HOMO–LUMO energy gaps (in eV) calculated at the B3LYP/6-311++G(d,p) level of theory. As can be seen from Figure 5, the SOMO-1 and SOMO-2 sets for diradical structures from **I-A** to **VI-A** are essentially nondisjoint type. Thus, based on the evidence presented above, it is expected that magnetic exchange coupling between radical sites would favor a predominantly antiferromagnetic character. This expectation is supported by the data displayed in Table 1, where **diradicals III-A**, **IV-A, and VI-A** correspond to negative *J* values (antiferromagnetic coupling) of −95.8, −923.0, and −1181.2 cm^–1^, respectively. At the same time, structures **I-A**, **II-A**, and **V-A represent** very small positive *J* values of 9.7, 21.8, and 15.1 cm^–1^, respectively, indicating ferromagnetic coupling. On the other hand, Figure 6 demonstrates that only structure **II-B** has SOMO-1 and SOMO-2 sets that are nondisjoint, while structures **I–B**, **III-B**, **IV-B**, **V-B**, and **VI-B** have SOMO-1 and SOMO-2 sets of disjoint type. Thus, large ferromagnetic coupling was observed for structures **III-B**, **IV-B**, **V-B, and VI-B** with *J* values of 1200.8, 350.3, 218.8, and 571.4 cm^–1^, respectively, which, indeed, is consistent with the arguments noted above that ferromagnetic coupling in diradicals is most likely associated with the presence of disjoint sets for SOMO-1 and SOMO-2. The data presented in Figures 5 and 6, in most cases, seem to support the numerical findings listed in Table 1 regarding the anticipated type of magnetic interaction in the diradicals considered here, which is very encouraging.

**Table 3:** SOMO-1 and SOMO-2 Energy Gaps and α-HOMO–LUMO Energy Gaps Calculated at the B3LYP/6-311++G(d,p) Level of Theory in Their High-Spin States

systems	SOMO-1 (au)	SOMO-2 (au)	LUMO (au)	SOMO–SOMO gap (eV)	HOMO–LUMO gap (eV)
IA	–0.20509	–0.19530	–0.11106	0.266	2.929
IB	–0.20402	–0.19356	–0.10895	0.285	2.302
IIA	–0.20470	–0.20353	–0.07766	0.032	3.425
IIB	–0.20490	–0.20309	–0.08241	0.049	3.284
IIIA	–0.20313	–0.20103	–0.12733	0.057	2.005
IIIB	–0.20330	–0.20015	–0.12711	0.086	1.988
IVA	–0.20273	–0.19464	–0.13031	0.220	1.751
IVB	–0.20486	–0.19971	–0.12280	0.140	2.092
VA	–0.20509	–0.20156	–0.13781	0.096	1.734
VB	–0.20260	–0.20002	–0.13441	0.070	1.785
VIA	–0.20174	–0.17267	–0.10864	0.791	1.742
VIB	–0.20211	–0.17205	–0.10047	0.818	1.948

Furthermore, we can examine the data in Table 3 displaying the SOMO–SOMO energy gaps Δ*E*_SOMO–SOMO_ as well as the HOMO–LUMO energy gaps Δ*E*_HL_ (in eV) obtained at the B3LYP/6-311++G(d,p) level of theory to determine whether the data in this table are also consistent with the calculated *J* values at the same level of theory. In general, it is considered that the SOMO–SOMO energy gap Δ*E*_SOMO–SOMO_ needs to be sufficiently small, Δ*E*_SOMO–SOMO_ < 1.5 eV^101^ or smaller. Zhang et al.^102^ explored a set of m-phenylene-linked diradicals to shed light on the effect of substitution on ground-state multiplicity, suggesting that Δ*E*_SOMO–SOMO_ < 0.19 eV, in order for two nonbonding electrons to occupy different degenerate (or near-degenerate) orbitals with parallel spin configuration (to minimize their electrostatic repulsion), thus resulting in a triplet ground electronic state, which is consistent with the well-known Hund’s rule.^103^ If the criterion of Δ*E*_SOMO–SOMO_**< 0**.19 eV is selected, then the B-type diradical structures **II-B**, **III-B**, **IV-B**, and **V-B**, with Δ*E*_SOMO–SOMO_ values of 0.049, 0.086, 0.140, and 0.070 eV, respectively, would be expected to have *ferromagnetic* coupling, while **I-B** with Δ*E*_SOMO–SOMO_ = 0.285 eV and **VI-B** with Δ*E*_SOMO–SOMO_ = 0.818 eV would be expected to have antiferromagnetic coupling. Thus, among the six B-type diradical structures, the criterion would correctly predict the correct type of magnetic coupling in four cases (67%), namely, for **I-B**, **III-B**, **IV-B**, and **V-B**, respectively. On the other hand, for the six A-type diradical structures (**I-A** to **VI-A**), the criterion Δ*E*_SS_ < 0.19 eV would yield correct predictions of ferromagnetic coupling in three of six cases (50%), namely for **II-A** (with Δ*E*_SOMO–SOMO_ = 0.032 eV and *J* = 21.8 cm^–1^), **V-A** (with Δ*E*_SOMO–SOMO_ = 0.096 eV and *J* = 15.09 cm^–1^), and **VI-A** (with Δ*E*_SOMO–SOMO_ = 0.791 eV and *J* = −1181.2 cm^–1^).

Overall, the analysis based on the shape and character of SOMO-1 and SOMO-2 sets, as well as SOMO–SOMO energy gaps, provides helpful insight regarding the characteristics of magnetic coupling in the diradical structures (A and B types) considered in this study in the majority of cases considered in the present work, which is encouraging. Notably, the Δ*E*_SOMO–SOMO_ values do not exceed 0.82 eV for any of our 12 diradical structures.

Finally, in order for the effective exchange coupling interaction to take place, it is essential to have *nonnegligible* LUMO contributions on atoms along possible exchange coupling pathways (ECPs) between singly occupied molecular orbitals, i.e., SOMO-1 and SOMO-2. As can be seen from Figures 5 and 6, indeed, all lowest-unoccupied molecular orbitals (LUMOs) are localized between the SOMO-1 and SOMO-2 orbitals for all 12 structures (**I-A** to **VI-A** and **I-B** to **VI-B**), which confirms our expectations (see ref (15)).

### 5.4 Spin Density Analysis

**Figure 7:**
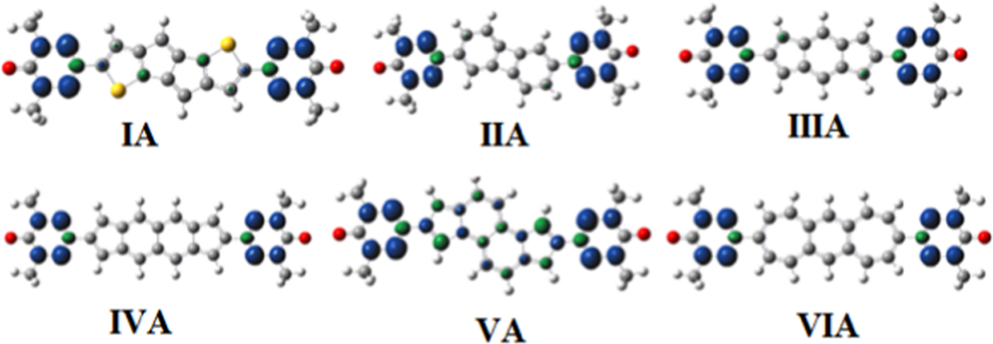
Spin density plots for A-type diradical structures at the B3LYP/6-311G++(d,p) level of theory in the high-spin state. The iso-value for the spin density plots is set as 0.001. The blue color represents spin-up densities, while the green color represents spin-down densities. Red, gray, blue, white, and yellow atoms represent the oxygen, carbon, nitrogen, hydrogen, and sulfur atoms, respectively.

**Figure 8:**
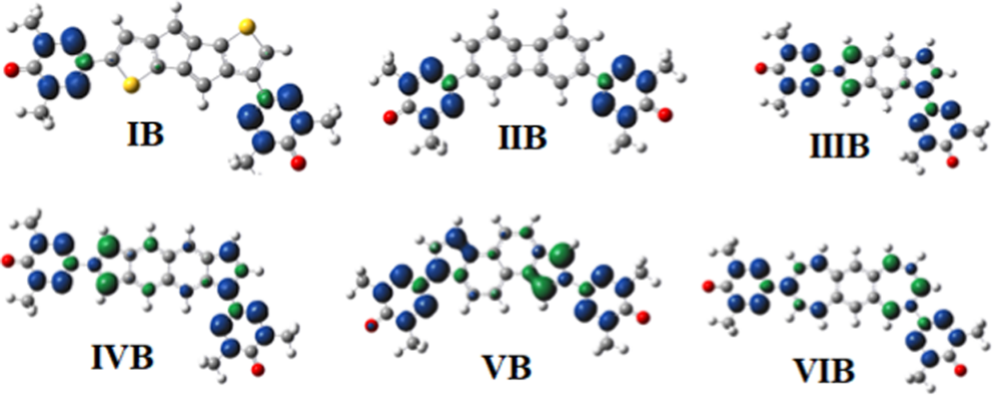
Spin density plots for B-type diradical structures at the B3LYP/6-311G++(d,p) level of theory in the high-spin state. The iso-value for the spin density plots is set as 0.001. The blue color represents spin-up densities, while the green color represents spin-down densities. Red, gray, blue, white, and yellow atoms represent the oxygen, carbon, nitrogen, hydrogen, and sulfur atoms, respectively.

In the discussion of spin exchange coupling for diradicals, the analysis of spin densities is of great importance.^17,18,22^ It is expected that nonnegligible spin densities would be observed on atoms located along the exchange coupling pathways if significant magnitudes for the magnetic exchange coupling constants are expected. For A-type diradical structures, Figure 7 indicates that nonnegligible contributions of spin densities on coupler atoms are observed for structures **I-A** and **V-A**, both having a ferromagnetic character of diradical. At the same time, the spin density plots presented in Figure 8 for the B-type diradicals considered in the present study show that especially large spin density contributions on coupler atoms are observed for structures **III-B**, **VI-B**, **V-B**, and **VI-B**, all having remarkably large ferromagnetic coupling constants *J*, i.e., 1200.8, 350.3, 218.8, and 571.4 cm^–1^ at the UB3LYP/6-311++G(d,p) level of theory, respectively. Clearly, the spin density plots presented in this study further support the exchange coupling character, where a large spin density contribution on coupler atoms favors ferromagnetic interactions in the diradicals considered in the present study.

## 6 Conclusions

A significant novel result regarding the diradical systems considered in the present study (12 diradical systems in total) is that even though the couplers used in this study are *antiaromatic*, the exchange coupling coefficients *J* exhibit remarkably large magnitudes for many of these systems, indicating ferromagnetic and antiferromagnetic coupling. For example, for some diradical systems, the *J* value reaches up to 1200 cm^–1^ (strong ferromagnetic coupling), while for other diradicals, *J* values reach −1181 cm^–1^ (strong antiferromagnetic coupling) at the UB3LYP/6-311++G(d,p) level of theory. At the UMN12SX/6-311++G(d,p) level of theory, for the diradical systems, *J* values vary from −568 cm^–1^ (antiferromagnetic coupling) to 289 cm^–1^ (ferromagnetic coupling).

On the basis of Nuclear Independent Chemical Shift data for NICS_*zz*_(+1) and the data for HOMA aromaticity indices, one can conclude that the diradicals **III-B**, **IV-B**, **V-B**, and **VI-B** achieve large positive exchange coupling constants *J* (indicating strong *ferromagnetic* coupling) by undergoing significant aromatic stabilization of their coupler systems. Furthermore, in antiaromatic couplers, the π-electrons circulate within the rings of a coupler and tend to gain π-electrons from outside (from radical moieties) to increase their aromaticity stability, thus facilitating π-conjugation. However, in *aromatic* couplers, π-electrons circulate within individual rings within the coupler and achieve a high degree of aromatic stability without the need to use the outside π-electrons from radical sites to enhance their stability. This phenomenon greatly influences the values of the magnetic coupling constant in diradicals coupled through aromatic or antiaromatic couplers.

In addition, spin density plots also support the exchange coupling character, where a large spin density contribution on coupler atoms is seen to favor ferromagnetic interactions in the diradicals considered here. For example, diradicals **III-B**, **IV-B**, **V-B**, and **VI-B**, having remarkably large ferromagnetic coupling constants *J*, namely 1200.8, 350.3, 218.8, and 571.4 cm^–1^, at the UB3LYP/6-311++G(d,p) level of theory, respectively, all have large spin density contributions on coupler atoms. Furthermore, for significant exchange coupling in diradicals, it is essential to have nonnegligible LUMO contributions to atoms along possible exchange coupling pathways (ECPs) between singly occupied molecular orbitals, i.e., SOMO-1 and SOMO-2. In this study, we find that all lowest-unoccupied molecular orbitals (LUMOs) are localized between the SOMO-1 and SOMO-2 orbitals for all 12 structures (**I-A** to **VI-A** and **I-B** to **VI-B**), which confirms our expectations. It is also interesting to note that for heteroatomic couplers, the exchange coupling constant *J* is smaller on average than that of homoatomic couplers, as expected.

The findings in this study offer new strategies for designing novel organic materials with interesting magnetic properties for practical applications.

## Supporting Information Available

The BS state molecular orbitals and spin density; the energy and spin *S*^2^ values of the diradicals; and optimized coordinates of all of the molecules (PDF)
