# Supplementary material for: Antiaromatic Molecules as Magnetic Couplers: A Computational Quest
Source: J Phys Chem A. 2024 Jan 24;128(5):815–28. doi: 10.1021/acs.jpca.3c05784 (PMC10860145; doi:10.1021/acs.jpca.3c05784)
Supplement: Supplementary file 1 — jp3c05784_si_001.pdf [file jp3c05784_si_001.pdf]

Supporting Information

Antiaromatic molecules as magnetic couplers: A computational quest

Suranjan Shil*[1], Debojit Bhattacharya[2], Anirban Misra[3], and Laimutis Bytautas[4]

[1]Manipal Centre for Natural Sciences (*Centre of Excellence*), Manipal Academy of Higher Education, Manipal-576104, India *Email: suranjan.shil@manipal.edu Phone: +91-820-2923580

[2]Kabi Sukanta High School, Siliguri-734010, India

[3]Department of Chemistry, University of North Bengal, Raja Rammohunpur, Siliguri 734013, India

[4] Department of Chemistry, Galveston College, 4015 Ave. Q, Galveston, Texas, 77550, USA

Table S1. The optimized UB3LYP level absolute energies (E, hartree), $\langle S^2 \rangle$, and intramolecular magnetic exchange coupling constants (J, cm^{-1}) using 6-311++G(d,p) basis set for all the diradicals (I-A to VI-A).

Diradical		UB3LYP/6-311++G(d,p) level of theory		
		High-Spin-State	Broken-Symmetry-State	(J, cm-1)
I-A	E	2156.83665	−2156.83661	9.72
	$\langle S^2 \rangle$	2.07	1.06	
II-A	E	−1361.62615	−1361.62605	21.79
	$\langle S^2 \rangle$	2.05	1.05	
III-A	E	−1361.63079	−1361.63118	−95.80
	$\langle S^2 \rangle$	2.06	1.16	
IV-A	E	−1515.30644	−1515.30874	−923.02
	$\langle S^2 \rangle$	2.05	1.50	
V-A	E	−1515.29213	−1515.29208	15.09
	$\langle S^2 \rangle$	2.22	1.50	
VI-A	E	−1516.49703	−1516.50063	−1181.17
	$\langle S^2 \rangle$	2.25	1.58	

Table S2. The optimized UB3LYP level absolute energies (E, hartree), $\langle S^2 \rangle$, and intramolecular magnetic exchange coupling constants (J, cm^{-1}) using 6-311++G(d,p) basis set for all the diradicals (I-B to VI-B).

Diradical		UB3LYP/6-311++G(d,p) level of theory		
		High-Spin-State	Broken-Symmetry-State	(J, cm^{-1})
I-B	E	−2156.83526	−2156.83531	−10.76
	$\langle S^2 \rangle$	2.05	1.06	
II-B	E	−1361.62611	−1361.62623	−26.72
	$\langle S^2 \rangle$	2.05	1.05	
III-B	E	−1361.63223	−1361.62511	1200.77
	$\langle S^2 \rangle$	2.35	1.05	
IV-B	E	−1515.31164	−1515.30992	350.29
	$\langle S^2 \rangle$	2.60	1.52	
V-B	E	−1515.29548	−1515.29429	218.75
	$\langle S^2 \rangle$	2.78	1.58	
VI-B	E	−1516.49507	−1516.49169	571.42
	$\langle S^2 \rangle$	2.47	1.17	

Table S3. The optimized unrestricted MN12SX level absolute energies (E, hartree), $\langle S^2 \rangle$, and intramolecular magnetic exchange coupling constants (J, cm^{-1}) using 6-311++G(d,p) basis set for all the diradicals (I-A to VI-A).

Diradical		MN12SX/6-311++G(d,p) level of theory		
		High-Spin-State	Broken-Symmetry-State	(J, cm^{-1})
I-A	E	−2155.67525	−2155.67525	0.00
	$\langle S^2 \rangle$	2.05	1.05	-4.56
II-A	E	−1360.53839	−1360.53831	18.00
	$\langle S^2 \rangle$	2.05	1.04	-20.80
III-A	E	−1360.54341	−1360.54360	-41.69
	$\langle S^2 \rangle$	2.05	1.06	153.38
IV-A	E	−1514.09955	−1514.10236	-218.08
	$\langle S^2 \rangle$	2.04	1.23	272.24
V-A	E	−1514.08518	−1514.08492	54.08
	$\langle S^2 \rangle$	2.11	1.07	193.02
VI-A	E	−1515.26836	−1515.27023	-568.12
	$\langle S^2 \rangle$	2.04	1.32	289.18

Table S4. The optimized MN12SX level absolute energies (E, hartree), $\langle S^2 \rangle$, and intramolecular magnetic exchange coupling constants (J, cm^{-1}) using 6-311++G(d,p) basis set for all the diradicals (I-B to VI-B).

Diradical		MN12SX/6-311++G(d,p) level of theory		
		High-Spin-State	Broken-Symmetry-State	(J, cm^{-1})
I-B	E	−2155.67471	−2155.67473	-4.56
	$\langle S^2 \rangle$	2.04	1.05	
II-B	E	−1360.53836	−1360.53846	-20.80
	$\langle S^2 \rangle$	2.04	1.05	
III-B	E	−1360.54463	−1360.54387	153.38
	$\langle S^2 \rangle$	2.18	1.10	
IV-B	E	−1514.10366	−1514.10229	272.24
	$\langle S^2 \rangle$	2.41	1.30	
V-B	E	−1514.08793	−1514.08699	193.02
	$\langle S^2 \rangle$	2.48	1.42	
VI-B	E	−1515.26543	−1515.26387	289.18
	$\langle S^2 \rangle$	2.26	1.07	

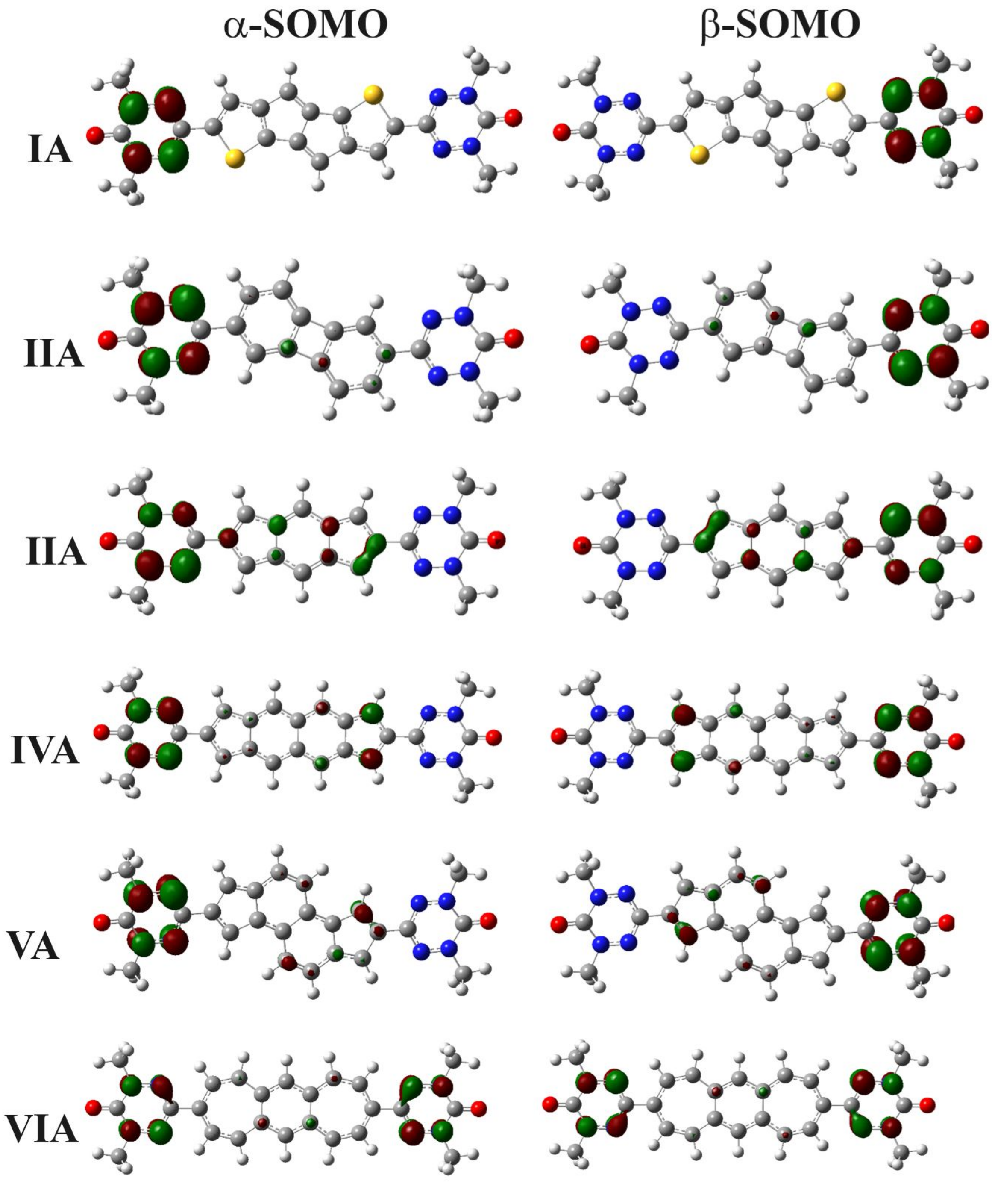

Figure S1. Molecular orbitals (α-SOMO, β-SOMO) for structures I-A to VI-A at B3LYP/6-311++G(d,p) level of theory in the BS spin state. Iso-value for MO plots is set to be 0.05. Red, grey, blue, white and yellow atoms represent oxygen, carbon, nitrogen, hydrogen and Sulphur atom respectively.

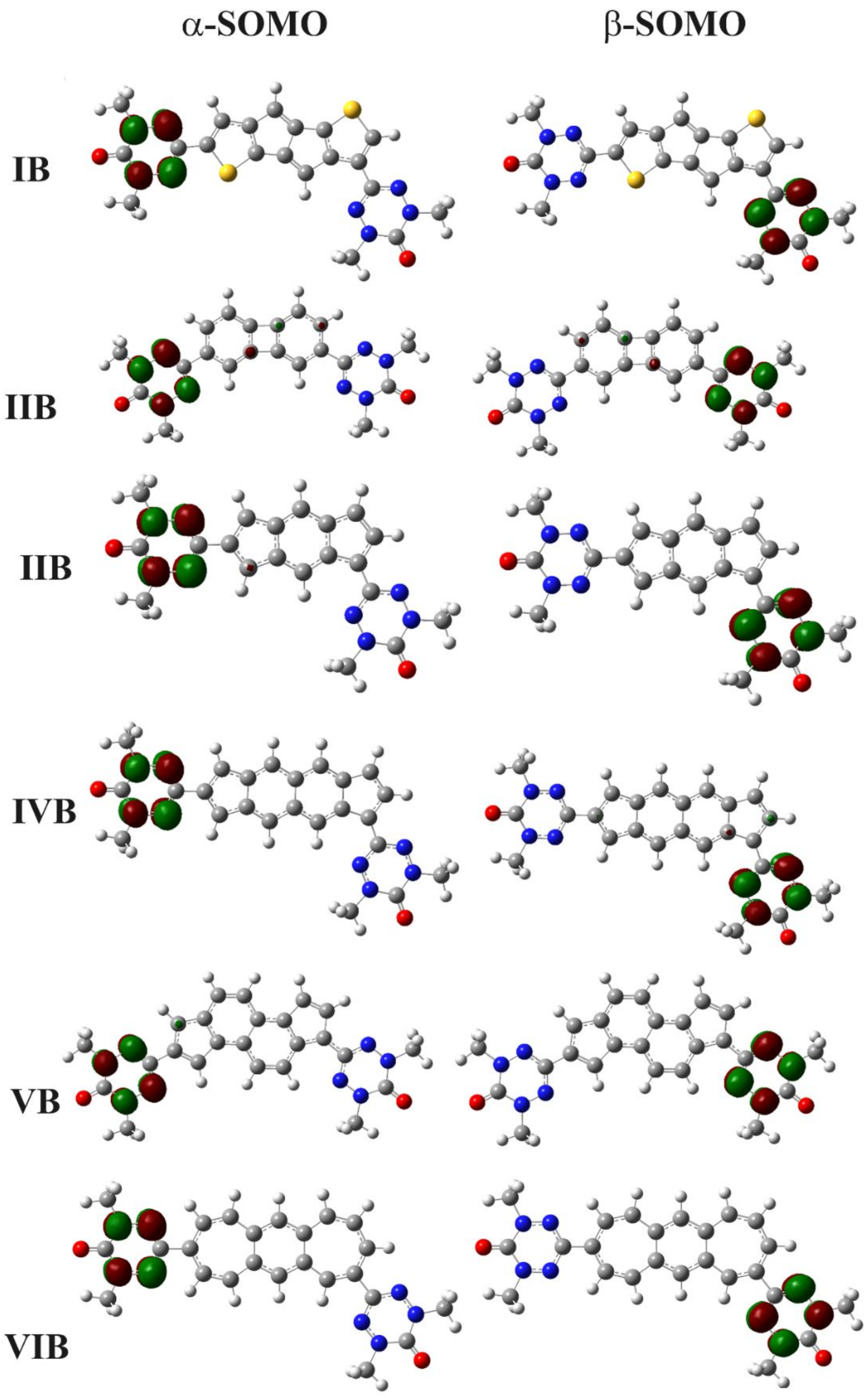

Figure S2. Molecular orbitals (α-SOMO, β-SOMO) for structures I-B to VI-B at B3LYP/6-311++G(d,p) level of theory in the BS spin state. Iso-value for MO plots is set to be 0.05. Red, grey, blue, white and yellow atoms represent oxygen, carbon, nitrogen, hydrogen and Sulphur atom respectively.

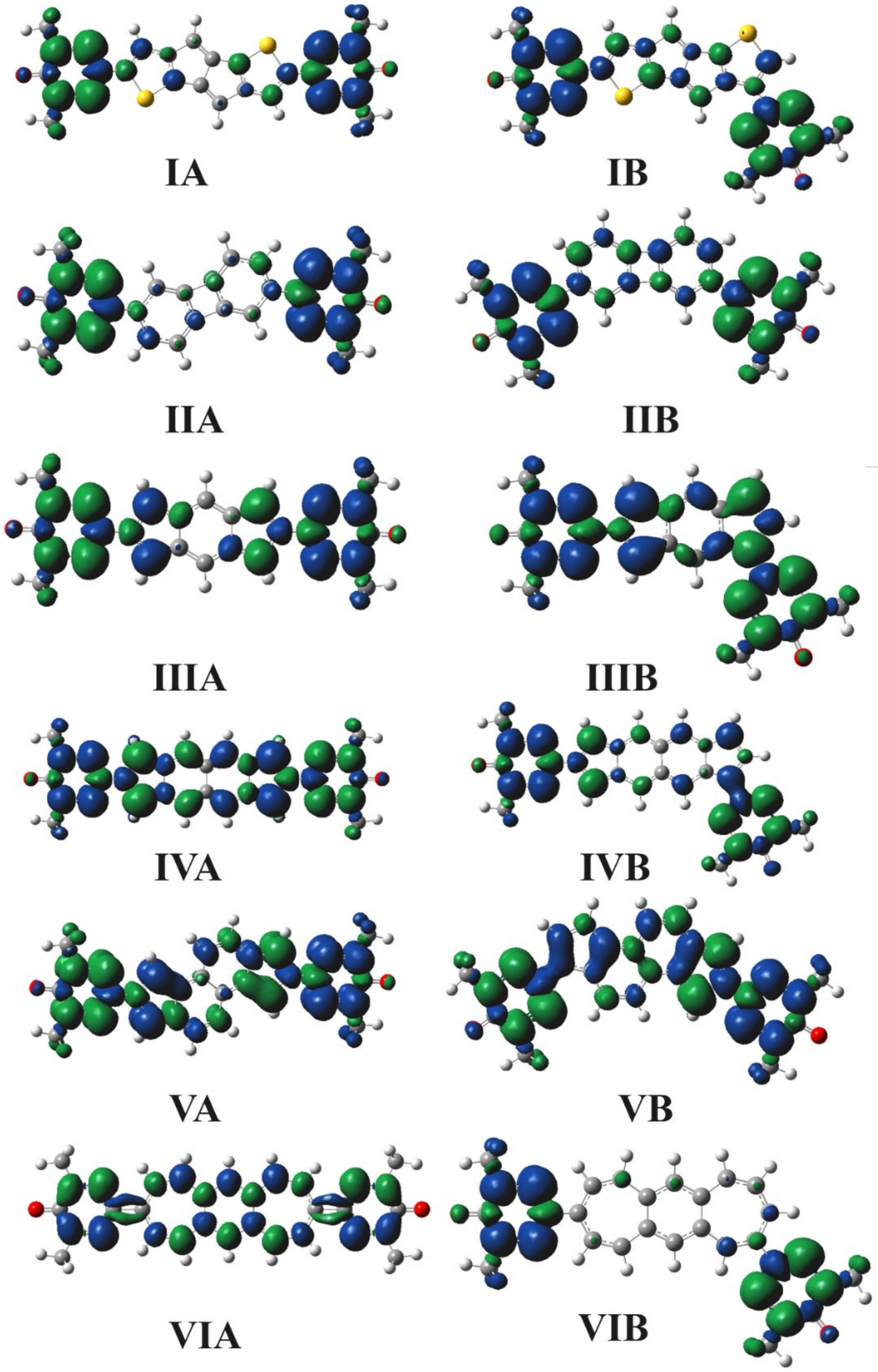

Figure S3. The spin density Plots of the diradical structures at B3LYP/6-311G++(d,p) level of theory in the BS spin state. Iso-value for spin density plots is set to be 0.001.The **blue color** represents **spin-up** densities while **green color** represents **spin-down** densities. Red, grey, blue, white and yellow atom represents oxygen, carbon, nitrogen, hydrogen and Sulphur atom respectively.

Optimized Coordinates of the molecules in different functionals

Functional-B3LYP

IA

C 0.66114600 -1.62694400 -0.01034600
C 1.96575300 -0.93176400 -0.00998300
C 1.74126100 0.44508300 -0.00982700
C 0.30853900 0.67616400 -0.01004600
C -0.30848100 -0.67613000 -0.01040500
C -0.66109100 1.62697700 -0.00986400
C -1.96569700 0.93179700 -0.01013500
C -1.74120200 -0.44504800 -0.01047500
H 0.53122300 -2.70094500 -0.01046100
H -0.53116800 2.70097800 -0.00950900
C -3.33401900 1.26935400 -0.00986600
H -3.74134400 2.27078400 -0.00943200
C -4.14513300 0.15005700 -0.00991000
C 3.33407100 -1.26932300 -0.00950500
H 3.74139800 -2.27075300 -0.00937900
C 4.14518500 -0.15002400 -0.00890500
S -3.20752800 -1.33820500 -0.01068200
S 3.20758600 1.33824200 -0.00929400
C 5.60358100 -0.10148900 -0.00788100
N 6.16765500 1.10785200 -0.00449600
C -5.60353600 0.10150100 -0.00925100
N -6.16753000 -1.10790300 -0.00593700
C -8.33216300 0.00589600 0.01479400
C 8.33241900 -0.00593100 0.01400300
N 6.24788200 -1.27256200 -0.01072700
N -6.24781900 1.27259600 -0.01237700
N 7.52620300 1.12606100 -0.00633300
N 7.60544400 -1.19105500 -0.00934600
N -7.60541800 1.19103800 -0.01135300
N -7.52610800 -1.12608900 -0.00853700
O -9.54951300 -0.03634900 0.04713600
O 9.54987400 0.03618000 0.04257300
C -8.30264900 2.47267700 0.01410400
H -9.36472200 2.29071900 -0.12295600
H -8.13034800 2.96885500 0.97222400
H -7.91374300 3.09942700 -0.78907600
C -8.13114500 -2.45361300 0.02576800
H -7.95803700 -2.91642600 1.00049200
H -9.19823300 -2.35130700 -0.14992400

H -7.67018800 -3.06659800 -0.74899100
C 8.13058900 2.45396600 0.02419000
H 7.93626900 2.92765600 0.98948200
H 9.20100900 2.34919100 -0.12794200
H 7.68703600 3.05862800 -0.76734900
C 8.30220300 -2.47301700 0.01269800
H 9.36627600 -2.28940200 -0.10504800
H 8.11348500 -2.97921100 0.96230100
H 7.92733200 -3.09150300 -0.80366500

IB

C -0.68366600 -2.75790400 0.00036000
C -1.73745100 -1.71855900 0.00040100
C -1.12840800 -0.46444000 0.00037300
C 0.31207200 -0.65344300 0.00026900
C 0.51518000 -2.12251800 0.00025600
C 1.51216200 -0.01792300 0.00020000
C 2.56617100 -1.05759000 0.00009100
C 1.95754600 -2.30703400 0.00017300
H -0.86578300 -3.82437500 0.00038700
H 1.71039900 1.04284100 0.00018400
C 3.99128800 -1.13422700 -0.00009900
C 4.42147600 -2.44637400 -0.00028800
C -3.14497200 -1.65067100 0.00044600
H -3.82180600 -2.49372100 0.00043100
C -3.60240300 -0.34598700 0.00035200
S 3.11077800 -3.58163700 0.00023800
S -2.27738900 0.81080400 0.00053900
C -4.98579900 0.11760000 0.00021400
N -5.18175500 1.43759700 0.00024200
C 4.92106200 0.00860800 -0.00024400
N 6.22687000 -0.27563300 -0.00049400
C 6.65242900 2.12397400 -0.00037900
C -7.57508200 0.98863600 -0.00019900
N -5.93819700 -0.82057000 0.00007600
N 4.38589900 1.22759300 -0.00014800
N -6.47895100 1.84275200 0.00011400
N -7.21585800 -0.35487000 0.00005900
N 5.27027900 2.26259900 -0.00024400
N 7.06317600 0.79445900 -0.00083900
O 7.42463500 3.06556200 0.00014200
O -8.73063400 1.37609100 -0.00090000
C 4.66943600 3.59216800 0.00026200
H 5.46670600 4.32982400 -0.00028800
H 4.04746500 3.70860900 -0.88938100
H 4.04874700 3.70861200 0.89081100
C 8.48611200 0.46982700 -0.00042200
H 9.05086500 1.39753600 -0.00466500
H 8.72288000 -0.11338200 0.89143900
H 8.72113700 -0.12083000 -0.88775500

C -6.67672400 3.28842600 -0.00029200
H -7.74414300 3.48918300 0.00096500
H -6.20933700 3.71669300 0.88837500
H -6.21166200 3.71608400 -0.89049800
C -8.24858100 -1.38571400 -0.00061700
H -8.13507600 -2.01038300 0.88738500
H -9.21991800 -0.89975800 0.00193900
H -8.13813500 -2.00666800 -0.89165500
H 5.44009300 -2.79877700 -0.00054400

IIA

C -2.29174800 0.69290100 -0.00005700
C -0.95500600 0.40284800 -0.00002700
C -1.29498700 -2.01391500 0.00002900
C -2.68079700 -1.72968000 -0.00000400
C -3.17767500 -0.42523600 -0.00004200
C 0.45597100 0.92908600 -0.00001600
C -0.45597100 -0.92908600 0.00001600
C 0.95500600 -0.40284800 0.00002700
C 1.29498700 2.01391500 -0.00002900
C 2.68079700 1.72968000 0.00000400
H 3.38973400 2.54702100 -0.00000300
C 3.17767500 0.42523600 0.00004200
C 2.29174800 -0.69290100 0.00005700
H -2.69011100 1.69803800 -0.00008700
H -3.38973400 -2.54702100 0.00000300
H 2.69011100 -1.69803800 0.00008700
C -4.64406400 -0.19135100 -0.00006800
C -7.34638700 0.25161100 -0.00014800
C 4.64406400 0.19135100 0.00006800
C 7.34638700 -0.25161100 0.00014800
N -5.43897700 -1.26487600 -0.00006300
N -6.77440100 -1.01582100 -0.00020400
N -6.40075200 1.26881500 -0.00009000
N -5.05472800 1.07656300 -0.00014300
N 5.05472800 -1.07656300 0.00014300
N 5.43897700 1.26487600 0.00006300
N 6.40075200 -1.26881500 0.00009000
N 6.77440100 1.01582100 0.00020400
O 8.54867700 -0.44761300 0.00029000
O -8.54867700 0.44761300 -0.00029000
C -7.62583000 -2.20102900 -0.00014900
H -7.41522500 -2.79672500 0.89003200
H -8.66331400 -1.87960200 -0.00140100
H -7.41336800 -2.79796300 -0.88903700
C -6.82749100 2.66413100 -0.00032300
H -6.43755400 3.16111100 -0.89065000
H -7.91330300 2.69191300 0.00102500

H -6.43522500 3.16193800 0.88849300
C 6.82749100 -2.66413100 0.00032300
H 6.43522500 -3.16193800 -0.88849300
H 7.91330300 -2.69191300 -0.00102500
H 6.43755400 -3.16111100 0.89065000
C 7.62583000 2.20102900 0.00014900
H 7.41336800 2.79796300 0.88903700
H 8.66331400 1.87960200 0.00140100
H 7.41522500 2.79672500 -0.89003200
H 0.94579200 3.03961700 -0.00006100
H -0.94579200 -3.03961700 0.00006100

IIB

C -1.90613900 -0.07064500 -0.00025500
C -0.75458100 -0.80783400 -0.00018400
C -1.91659000 -2.95517800 -0.00012600
C -3.11630700 -2.20613500 -0.00020800
C -3.12651000 -0.81034400 -0.00028900
C 0.75458100 -0.80783400 -0.00011800
C -0.75169700 -2.23015000 -0.00011800
C 0.75169700 -2.23015000 -0.00003800
C 1.90613900 -0.07064500 -0.00007900
C 3.12650900 -0.81034300 0.00004700
C 3.11630700 -2.20613500 0.00012400
C 1.91658900 -2.95517800 0.00008700
H -1.92920200 1.01038000 -0.00030800
H -4.06579500 -2.72506700 -0.00022600
H 1.94675200 -4.03837800 0.00015100
C -4.41934500 -0.07978300 -0.00039300
C -6.79694300 1.27751500 -0.00004500
C 4.41934500 -0.07978300 0.00010100
C 6.79694400 1.27751500 0.00018600
N -5.53856800 -0.80897400 -0.00021000
N -6.70301200 -0.10996000 -0.00009000
N -5.55603100 1.90113500 0.00000100
N -4.36181600 1.25156000 -0.00023900
N 5.53856800 -0.80897400 0.00022500
N 4.36181600 1.25156000 -0.00004300
N 6.70301200 -0.10996000 0.00017300
N 5.55603100 1.90113500 0.00004300
O 7.85562800 1.88051800 0.00013200
O -7.85562800 1.88051800 0.00034400
C -7.91430900 -0.92355100 0.00023700
H -7.92447100 -1.55567100 0.89029100
H -8.77453300 -0.26036500 -0.00052700
H -7.92387600 -1.55705500 -0.88882200
C -5.46934200 3.35753100 0.00016200
H -4.93001200 3.68760100 -0.88984700

H -6.47733200 3.76231600 0.00111600
H -4.92841300 3.68721800 0.88932800
C 7.91430900 -0.92355200 0.00036700
H 7.92367200 -1.55720600 -0.88858500
H 8.77453300 -0.26036600 -0.00069600
H 7.92467500 -1.55552000 0.89052700
C 5.46934300 3.35753100 -0.00020300
H 4.92845600 3.68746600 0.88889800
H 6.47733200 3.76231600 0.00059600
H 4.92997000 3.68735300 -0.89027800
H 1.92920200 1.01038000 -0.00013000
H -1.94675200 -4.03837800 -0.00007200
H 4.06579500 -2.72506700 0.00021700

IIIA

**C**	**2.53285300**	**1.11656800**	**0.00436400**
**C**	**1.20420700**	**0.70845500**	**0.00464100**
**C**	**1.19495500**	**-0.74659400**	**0.00436000**
**C**	**2.56829100**	**-1.17753900**	**0.00389400**
**C**	**3.37641800**	**-0.05219500**	**0.00388600**
**C**	**-0.00937600**	**1.44607300**	**0.00499300**
**C**	**0.00939300**	**-1.44612400**	**0.00439300**
**C**	**-1.20419200**	**-0.70850500**	**0.00474500**
**C**	**-1.19493600**	**0.74654200**	**0.00505700**
**C**	**-2.56827000**	**1.17749300**	**0.00524200**
**H**	**-2.91500000**	**2.20061200**	**0.00545100**
**C**	**-3.37640300**	**0.05215400**	**0.00499300**
**C**	**-2.53284300**	**-1.11661300**	**0.00473800**
**H**	**0.00737200**	**2.53208200**	**0.00518600**
**H**	**2.89784000**	**2.13396500**	**0.00442800**
**H**	**2.91503000**	**-2.20065600**	**0.00357700**
**H**	**-0.00735600**	**-2.53213500**	**0.00414000**
**H**	**-2.89783500**	**-2.13401000**	**0.00450000**
**C**	**4.83959300**	**-0.02852000**	**0.00319200**
**C**	**7.57382200**	**0.02378400**	**-0.00503300**

**C**	**-4.83957900**	**0.02850400**	**0.00460700**
**C**	**-7.57375200**	**-0.02372700**	**-0.00544000**
**N**	**5.46977700**	**-1.20873000**	**0.00253100**
**N**	**6.82762700**	**-1.15005100**	**0.00171900**
**N**	**6.78436800**	**1.16635500**	**0.00329000**
**N**	**5.42378700**	**1.17068700**	**0.00328300**
**N**	**-5.46971100**	**1.20875400**	**0.00388500**
**N**	**-5.42380200**	**-1.17068500**	**0.00511400**
**N**	**-6.82756900**	**1.15007400**	**0.00487900**
**N**	**-6.78439600**	**-1.16633500**	**0.00430500**
**O**	**-8.79224900**	**-0.04553900**	**-0.01854100**
**O**	**8.79234700**	**0.04580800**	**-0.01493700**
**C**	**7.50306700**	**-2.44343500**	**-0.00719700**
**H**	**7.23894200**	**-2.98503300**	**-0.91782000**
**H**	**8.57505000**	**-2.27281400**	**0.03022000**
**H**	**7.17951300**	**-3.02216900**	**0.85962100**
**C**	**7.40783500**	**2.48528100**	**-0.00575200**
**H**	**7.05435300**	**3.05418500**	**0.85585700**
**H**	**8.48540000**	**2.35731200**	**0.04159100**
**H**	**7.13140800**	**3.01301700**	**-0.92100400**
**C**	**-7.50327600**	**2.44328400**	**-0.00871300**
**H**	**-7.26751300**	**2.96918500**	**-0.93645800**
**H**	**-8.57372900**	**2.27395000**	**0.06307900**
**H**	**-7.15308300**	**3.03638900**	**0.83745200**
**C**	**-7.40787900**	**-2.48523800**	**-0.00597000**
**H**	**-7.05521800**	**-3.05449300**	**0.85574500**
**H**	**-8.48549000**	**-2.35726400**	**0.04038300**
**H**	**-7.13059900**	**-3.01262200**	**-0.92116800**

IIIB

C	-1.61908400	0.36213900	-0.00012400
C	-0.47322200	-0.49567200	-0.00001900
C	-0.95749300	-1.85290400	0.00001000
C	-2.37318200	-1.79783600	-0.00007800
C	-2.77209600	-0.43667800	-0.00017100
C	0.88956800	-0.21352600	0.00007500
C	-0.05940700	-2.93017200	0.00013400
C	1.30264600	-2.64863200	0.00024000
C	1.79034600	-1.29195000	0.00021600
C	3.22342300	-1.36708300	0.00023700
C	3.59277500	-2.74586400	0.00025300
C	2.44562900	-3.51859400	0.00027600
H	1.25713800	0.80439700	0.00006800
H	-1.60901300	1.44268100	-0.00016800
H	-3.04985400	-2.64060300	-0.00009400
H	-0.41693000	-3.95572800	0.00014700
H	2.40878200	-4.59996600	0.00033000
C	-4.15272100	0.04464800	-0.00027600
C	-6.73691500	0.94264000	-0.00002900
C	4.18684600	-0.27745500	0.00024000
C	6.03651700	1.74224700	-0.00010900
N	-5.11744000	-0.87921100	-0.00030500
N	-6.39156900	-0.40300400	-0.00044200
N	-5.63116900	1.78452400	-0.00033600
N	-4.33630900	1.36886000	-0.00030800
N	3.72205400	0.97962900	0.00061300
N	5.48178300	-0.62861100	-0.00013500
N	4.66250100	1.95910800	0.00047800
N	6.37357900	0.39273200	0.00005900
O	6.85797800	2.64014100	-0.00058500

**O**	**-7.88844500**	**1.34315000**	**0.00043700**
**C**	**-7.43348400**	**-1.42410500**	**0.00001400**
**H**	**-7.32936600**	**-2.04628100**	**0.89104200**
**H**	**-8.40049600**	**-0.92952700**	**-0.00291800**
**H**	**-7.32572700**	**-2.05021500**	**-0.88776700**
**C**	**-5.81601800**	**3.23163000**	**0.00008000**
**H**	**-5.34406600**	**3.65601300**	**-0.88814400**
**H**	**-6.88140500**	**3.44297900**	**-0.00197200**
**H**	**-5.34781500**	**3.65532400**	**0.89065500**
**C**	**4.13934800**	**3.32206200**	**0.00066400**
**H**	**3.52596600**	**3.47319900**	**0.89086400**
**H**	**4.97744000**	**4.01284300**	**0.00058400**
**H**	**3.52569100**	**3.47337800**	**-0.88932100**
**C**	**7.77748300**	**-0.00959700**	**-0.00097700**
**H**	**7.98079900**	**-0.60497900**	**-0.89283900**
**H**	**8.39275000**	**0.88535800**	**0.00286100**
**H**	**7.97954000**	**-0.61211500**	**0.88629400**
**H**	**4.61051800**	**-3.10306700**	**0.00026500**

IVA

C -6.06358400 -0.00013500 -0.00046600
C -8.79795000 0.00014600 -0.00014900
C 6.06358200 0.00013200 0.00090100
C 8.79794700 -0.00013800 -0.00062400
N -6.67019600 1.18934000 -0.00078900
N -8.02977000 1.15847500 -0.00099500
N -8.02999200 -1.15836800 -0.00030900
N -6.67044600 -1.18952300 -0.00022600
N 6.67044300 1.18952300 0.00160300
N 6.67020000 -1.18933700 0.00021000
N 8.02999000 1.15837000 0.00158300
N 8.02977200 -1.15847200 0.00041300
O 10.01656200 -0.00024000 -0.00285000
O -10.01656600 0.00025400 0.00047300
C -8.67866900 2.46525700 -0.00070200
H -8.37117100 3.02095200 -0.88857100
H -9.75447700 2.31578800 -0.00362100

H -8.37592400 3.01865600 0.89028600
C -8.67918400 -2.46500600 0.00042100
H -8.37493200 -3.01884300 0.89060600
H -9.75496000 -2.31529700 -0.00056400
H -8.37342900 -3.02040000 -0.88825600
C 8.67918800 2.46500500 -0.00006900
H 8.36455900 3.02457500 0.88276600
H 9.75490800 2.31534700 0.01185100
H 8.38386700 3.01461300 -0.89596700
C 8.67867800 -2.46524700 -0.00204600
H 8.38036500 -3.01541300 -0.89657800
H 9.75446100 -2.31580700 0.00645700
H 8.36678000 -3.02414500 0.88221400
C -2.42952200 -0.72899900 0.00020500
C -1.22874300 -1.42244600 0.00050000
C 0.00002600 -0.72758900 0.00050600
C -0.00002700 0.72758300 0.00017900
C -1.22861600 1.42229800 -0.00011600
C -2.42956500 0.72873300 -0.00010900
C 1.22861500 -1.42230400 0.00081100
C 1.22874100 1.42244100 0.00016800
C 2.42952100 0.72899400 0.00047300
C 2.42956300 -0.72873800 0.00078700
C 3.77964300 -1.14976100 0.00097200
C 4.59726800 0.00027000 0.00083800
C 3.77983800 1.15008800 0.00055700
H 1.21783100 -2.50865800 0.00103000
H -1.21782100 -2.50879800 0.00075200
H -1.21783300 2.50865200 -0.00036100
H 1.21781700 2.50879300 -0.00006000
H 4.13825300 -2.16849200 0.00117600
H 4.13834800 2.16885300 0.00040400
C -3.77964400 1.14975600 -0.00038400
C -3.77984000 -1.15009400 0.00015700
C -4.59727000 -0.00027500 -0.00025300
H -4.13834900 -2.16885800 0.00034900
H -4.13825400 2.16848800 -0.00067000

IVB

C -5.38716700 -0.21771400 -0.00037100
C -8.02619500 -0.93346800 0.00021600
C 5.32420800 0.11456700 0.00004800
C 7.00664500 -2.04892100 0.00001400
N -6.28362700 0.77090000 -0.00034000
N -7.58790800 0.38535400 -0.00086100
N -6.98152000 -1.85017400 -0.00021000
N -5.66113900 -1.52413000 -0.00048800
N 6.64533700 0.35986800 0.00006900

N 4.76038700 -1.10229500 0.00001300
N 7.45105800 -0.73181000 0.00000900
N 5.62013500 -2.15412300 0.00001800
O 7.75326400 -3.01105600 0.00002600
O -9.20239800 -1.25257800 0.00116100
C -8.55593100 1.47690000 0.00049400
H -8.39956300 2.09831700 -0.88323100
H -9.55513900 1.05123800 -0.00981500
H -8.41339200 2.08566300 0.89548600
C -7.26557800 -3.28119900 0.00015900
H -6.82545300 -3.73641900 0.88944000
H -8.34299900 -3.41856200 0.00066700
H -6.82625700 -3.73670100 -0.88937700
C 8.88258600 -0.44440900 0.00009600
H 9.13337100 0.13575700 0.88995500
H 9.42354100 -1.38617200 -0.00057800
H 9.13315200 0.13696000 -0.88902800
C 4.98957300 -3.47034500 -0.00001400
H 4.36598100 -3.57244900 -0.89023000
H 5.76964100 -4.22607900 0.00007500
H 4.36581600 -3.57240200 0.89009100
C -1.67928000 0.04032800 -0.00015200
C -0.34380700 -0.31537600 -0.00009600
C 0.66260300 0.68271700 -0.00003100
C 0.28119400 2.07765700 -0.00001300
C -1.08954200 2.42779600 -0.00007500
C -2.06143600 1.44200400 -0.00014900
C 2.03247600 0.32762300 0.00000600
C 1.29331600 3.06831500 0.00005300
C 2.62473500 2.70549400 0.00009100
C 3.01019700 1.30783200 0.00005900
C 4.45115200 1.27278500 0.00008600
C 4.91131700 2.61864900 0.00015500
C 3.82816500 3.48396200 0.00014600
H 2.31111100 -0.71833800 -0.00001300
H -0.04599900 -1.35976200 -0.00010500
H -1.36459500 3.47854300 -0.00006800
H 1.00532000 4.11556300 0.00006900
H 3.87789400 4.56434400 0.00018400
C -3.48233400 1.49174000 -0.00024500
C -2.88074100 -0.72508100 -0.00022500
C -3.96943500 0.16728700 -0.00028100
H -2.95672100 -1.80246500 -0.00024000
H -4.09515600 2.38113700 -0.00027400
H 5.95311800 2.90091900 0.00019400

VA

C 5.54585700 -0.12492900 0.00016800

C 8.25479000 0.24346600 -0.00017900
C -5.54586000 0.12494100 0.00011000
C -8.25478900 -0.24348700 -0.00000800
N 6.30763900 -1.22009300 0.00012200
N 7.65017800 -1.00790100 0.00017500
N 7.33712500 1.29014400 0.00008700
N 5.98727600 1.14034400 0.00018200
N -5.98726300 -1.14034300 0.00003300
N -6.30765300 1.22009000 0.00008000
N -7.33710500 -1.29015900 -0.00001700
N -7.65019500 1.00788100 0.00005500
O -9.46128200 -0.40861400 -0.00031700
O 9.46128700 0.40857000 -0.00057900
C 8.46904000 -2.21586500 -0.00026200
H 8.24335200 -2.80500300 -0.89115200
H 9.51498800 -1.92319500 0.00216400
H 8.23977100 -2.80766900 0.88789700
C 7.80691100 2.67203700 -0.00013200
H 7.42973900 3.18084000 0.88890700
H 8.89298000 2.66739400 0.00047200
H 7.43078900 3.18031900 -0.88992700
C -7.80687900 -2.67205600 -0.00021400
H -7.43019100 -3.18072300 0.88911400
H -8.89294800 -2.66742200 -0.00017300
H -7.43026400 -3.18046900 -0.88972000
C -8.46906600 2.21583800 -0.00018600
H -8.24228100 2.80575000 -0.89027200
H -9.51501400 1.92315700 0.00076300
H -8.24090300 2.80687500 0.88878200
C 2.09460500 -1.45638600 0.00012800
C 1.84042700 -0.01874800 0.00011100
C 0.55574100 0.48585500 0.00009200
C -0.55574100 -0.48582900 0.00009100
C -0.27328800 -1.90311300 0.00010800
C 1.00067900 -2.37696000 0.00012700
C 0.27328700 1.90313000 0.00007500
C -1.84042000 0.01877400 0.00006900
C -2.09460100 1.45640400 0.00005300
C -1.00068800 2.37697800 0.00005900
C -3.45515400 1.64378900 0.00002900
C -4.10033600 0.33038100 0.00007900
C -3.13235200 -0.64366100 0.00004600
H 1.11114500 2.58870900 0.00007500
H -1.11115000 -2.58868800 0.00010300
H 1.19450000 -3.44424500 0.00013900
H -1.19450700 3.44426300 0.00004400
H -3.98633300 2.58420400 0.00001900
H -3.31305100 -1.70685500 0.00004800
C 3.45514200 -1.64377600 0.00013900
C 4.10033300 -0.33035500 0.00014800

C 3.13235100 0.64368500 0.00011500
H 3.31305000 1.70687900 0.00010500
H 3.98632800 -2.58418800 0.00015100

VB

C 5.17329200 0.16633400 0.00197700
C 7.62628400 1.37523300 0.00028000
C -5.12809000 -0.03288200 -0.00657100
C -7.25194400 1.69727000 0.00341700
N 6.24354600 -0.63103600 0.00386000
N 7.44898500 -0.00346800 0.00411200
N 6.42561100 2.07514900 0.00163200
N 5.19133200 1.50418100 0.00057400
N -6.36190100 -0.56866300 -0.00571700
N -4.85101600 1.28235200 -0.00927100
N -7.39103500 0.31383900 -0.01160000
N -5.92326900 2.11260100 -0.00862700
O -8.19430000 2.46662900 0.02066400
O 8.71985700 1.91327900 -0.00299600
C 8.60871900 -0.88894600 0.00317400
H 8.58727400 -1.51263400 -0.89265000
H 9.50745800 -0.27918700 0.01535600
H 8.57343100 -1.52969000 0.88618500
C 6.43180600 3.53421200 -0.00374200
H 5.89060000 3.89875900 0.87119000
H 7.46288200 3.87474000 0.02080900
H 5.93606700 3.89530500 -0.90704300
C -8.72323400 -0.28483900 0.01058800
H -8.88917800 -0.77983000 0.97010600
H -9.45856800 0.50151300 -0.13302000
H -8.78892700 -1.02208900 -0.78995700
C -5.60521100 3.53791900 0.00219000
H -4.97570600 3.76947800 -0.85836600
H -6.53384800 4.09893800 -0.04720700
H -5.06620900 3.78383600 0.91933100
C 2.25816600 -2.14732900 0.00210400
C 1.59797000 -0.88506600 -0.00008200
C 0.20187800 -0.79005700 -0.00150400
C -0.55344600 -2.04398100 -0.00053900
C 0.14210000 -3.29258100 0.00165900
C 1.51329500 -3.35058800 0.00291900
C -0.49950500 0.45316100 -0.00378100
C -1.94995500 -1.95074100 -0.00180200
C -2.61822500 -0.69601400 -0.00402600
C -1.87013400 0.50867900 -0.00502300
C -4.02508200 -0.96597400 -0.00467500

C -4.20017900 -2.40505500 -0.00270600
C -2.97346800 -2.99962800 -0.00106700
H 0.07460800 1.37165100 -0.00449500
H -0.43587700 -4.20832600 0.00230800
H 2.02258100 -4.30840900 0.00456200
H -2.38538500 1.45871700 -0.00670400
H -2.78616800 -4.06371500 0.00051800
C 3.65187800 -1.90467700 0.00310400
C 3.86600500 -0.48621400 0.00155900
C 2.63216400 0.14335500 -0.00034300
H 2.47837900 1.21164900 -0.00175700
H 4.43620900 -2.64796000 0.00471300
H -5.16036700 -2.89623800 -0.00261700

VIA

C -5.90521100 0.00000000 -0.00058900
N -6.52165700 -1.18561500 -0.00090900
C 5.90074600 0.00000200 -0.00009600
N 6.52032800 -1.18780700 0.00059800
C 8.64668600 -0.00000500 0.00045900
C -8.64923700 -0.00000200 0.00064100
N -6.52165700 1.18561300 0.00010500
N 6.52033500 1.18780900 -0.00075500
N -7.87923500 -1.15592900 -0.00048100
N -7.87923700 1.15592500 0.00017900
N 7.87769500 1.15651200 0.00017100
N 7.87768900 -1.15651600 -0.00026400
O 9.86575600 -0.00001300 0.00111100
O -9.86855700 -0.00000200 0.00175200
C 8.52610200 2.46378100 -0.00085200
H 9.60190700 2.31448500 0.00718000
H 8.22753300 3.01474100 -0.89477700
H 8.21462000 3.02150600 0.88429100
C 8.52609800 -2.46378300 0.00195400
H 8.20998000 -3.02414700 -0.87981400
H 9.60185100 -2.31453600 -0.01186500
H 8.23221700 -3.01205700 0.89913500
C -8.52646400 -2.46354900 -0.00028400
H -8.22189500 -3.01693500 0.89008600
H -9.60241000 -2.31508200 -0.00183900
H -8.21931300 -3.01814600 -0.88897300
C -8.52646900 2.46354200 0.00187000
H -9.60240200 2.31508800 -0.00403600
H -8.22533200 3.01449700 0.89494900
H -8.21590200 3.02055600 -0.88407800
C 4.42329900 0.00000400 -0.00017800
C 3.76051000 1.24553600 -0.00017700
C 3.76051200 -1.24552600 -0.00022900

C 2.41600600 1.54444300 -0.00021600
H 4.41818000 2.10671200 -0.00011400
C 2.41600600 -1.54443400 -0.00031300
H 4.41818100 -2.10670200 -0.00021300
C 1.24620300 0.72181800 -0.00030300
H 2.19411100 2.60794200 -0.00016900
C 1.24620400 -0.72181000 -0.00037100
H 2.19411100 -2.60793300 -0.00035700
C -4.41912000 0.00000100 -0.00059200
C -3.75936200 1.24415800 -0.00043400
C -3.75936000 -1.24415900 -0.00072100
C -2.41353700 1.54480200 -0.00038300
H -4.41758100 2.10504800 -0.00036000
C -2.41353600 -1.54480000 -0.00070000
H -4.41757700 -2.10504900 -0.00085300
C -1.24449000 0.72233200 -0.00041400
H -2.19242200 2.60838500 -0.00028500
C -1.24448800 -0.72232700 -0.00054000
H -2.19241700 -2.60838200 -0.00081300
C 0.00102100 1.36610600 -0.00031500
C 0.00102200 -1.36609900 -0.00050100
H 0.00114600 2.45098800 -0.00024200
H 0.00114700 -2.45098100 -0.00057500

VIB

C 5.40671400 0.02298000 0.00005800
N 5.16319800 1.33425000 0.00039200
C -5.45705600 0.23712600 0.00028500
N -5.76293700 1.54276900 0.00030900
C -8.11802600 0.92054500 -0.00034000
C 7.57103700 1.70063200 -0.00013000
N 6.61430900 -0.53881300 -0.00023400
N -6.35440000 -0.75977700 0.00023200
N 6.25339900 2.14499300 0.00087100
N 7.67238600 0.31564500 -0.00059200
N -7.66117200 -0.39082800 0.00035700
N -7.08575000 1.84935700 0.00016700
O -9.29901200 1.22389800 -0.00110400
O 8.53247800 2.44757800 -0.00046100
C -8.61479400 -1.49531300 -0.00046100
H -9.61954500 -1.08276100 0.00450200
H -8.46088600 -2.10523000 -0.89273300
H -8.45421400 -2.11141000 0.88627900
C -7.38900900 3.27669600 -0.00041800
H -6.95782400 3.73745400 -0.89124900
H -8.46821900 3.39938600 0.00179100
H -6.95384600 3.73867900 0.88779500
C 5.96332400 3.57498600 0.00048600

H 5.37657900 3.82468900 0.88651700
H 6.90461700 4.11690900 0.00760100
H 5.38893100 3.82766100 -0.89289800
C 8.98417400 -0.32307000 -0.00088700
H 9.74460500 0.45252200 -0.00385400
H 9.08336500 -0.94604900 0.89011500
H 9.08050400 -0.95032300 -0.88914800
C -4.02938300 -0.12931100 0.00030900
C -3.69592900 -1.50204100 0.00037700
C -3.07497300 0.91588000 0.00027800
C -2.46891600 -2.12516700 0.00034800
H -4.54726200 -2.17244000 0.00046600
C -1.70170700 0.87205700 0.00025600
H -3.49956900 1.91276800 0.00027700
C -1.12977000 -1.61453000 0.00018900
H -2.51636500 -3.21050700 0.00043100
C -0.77105200 -0.22146000 0.00018800
H -1.22060500 1.84610400 0.00025400
C 4.51011200 -2.27807800 -0.00009100
C 3.58982000 -3.33280200 -0.00008700
C 4.22467500 -0.90012800 0.00003200
C 2.21016500 -3.32445600 -0.00000400
H 4.03677400 -4.32358400 -0.00015500
C 2.97155500 -0.29867700 0.00012200
C 1.28330600 -2.23334900 0.00005900
H 1.74024300 -4.30359600 0.00000100
C 1.64649600 -0.83686400 0.00008700
H 2.99824100 0.78335400 0.00022000
C -0.08192400 -2.54762600 0.00011800
C 0.59388600 0.09409900 0.00016700
H -0.35080000 -3.59880900 0.00012600
H 0.86143500 1.14532700 0.00020800
H 5.55653700 -2.54790000 -0.00017700

MN12SX

IA

C 0.65169100 -1.62472400 -0.01632000
C 1.95395800 -0.93911500 -0.01663900
C 1.73692000 0.43537400 -0.01645900
C 0.31002300 0.67111300 -0.01643800
C -0.31003500 -0.67106400 -0.01634800
C -0.65170200 1.62477200 -0.01653200
C -1.95396700 0.93916300 -0.01674300
C -1.73693400 -0.43532500 -0.01637800
H 0.51598700 -2.70256600 -0.01614800
H -0.51599800 2.70261400 -0.01651300

C -3.31770600 1.27656600 -0.01827700
H -3.73419300 2.27913100 -0.01886600
C -4.11792500 0.15419000 -0.01873700
C 3.31769800 -1.27651700 -0.01806200
H 3.73420300 -2.27907500 -0.01847800
C 4.11791300 -0.15413700 -0.01859100
S -3.19120300 -1.31692200 -0.01808900
S 3.19118400 1.31697000 -0.01824200
C 5.57181000 -0.10287000 -0.01999600
N 6.12527000 1.10596100 -0.01361500
C -5.57182200 0.10290600 -0.02029100
N -6.12532100 -1.10584300 -0.01422400
C -8.27558300 0.00237200 0.02553700
C 8.27562100 -0.00240700 0.02546900
N 6.21134700 -1.26996700 -0.02832800
N -6.21131300 1.27010000 -0.02846800
N 7.47996200 1.12009600 -0.02198300
N 7.56520400 -1.18088500 -0.03440300
N -7.56509700 1.18080600 -0.03484900
N -7.48008300 -1.12019700 -0.02278400
O -9.48493600 -0.04780400 0.09952500
O 9.48498500 0.04885500 0.09862600
C -8.27544900 2.43890000 0.02760300
H -9.26064000 2.31671000 -0.42617900
H -8.40025600 2.76527900 1.06860800
H -7.68692500 3.18205100 -0.51493300
C -8.10594500 -2.42222300 0.05197600
H -8.37945300 -2.66641600 1.08680700
H -9.01258800 -2.42620900 -0.55792800
H -7.38505000 -3.15295000 -0.31872700
C 8.10693300 2.42168900 0.05188600
H 8.39555200 2.65920800 1.08411200
H 9.00491700 2.43013000 -0.57085700
H 7.38056600 3.15427700 -0.30399000
C 8.27449400 -2.43956500 0.02729900
H 9.26535600 -2.31351100 -0.41266700
H 8.38522400 -2.77409200 1.06730000
H 7.69347500 -3.17885500 -0.52862800

IB

C 0.67882700 -2.75711000 -0.00018600
C 1.73080500 -1.72577000 -0.00015400
C 1.12728900 -0.47250200 -0.00008200
C -0.30823100 -0.65762500 -0.00006200
C -0.51469100 -2.11896300 -0.00012800
C -1.50232700 -0.01777400 -0.00000600
C -2.55378400 -1.05068300 -0.00002500
C -1.95204000 -2.29998300 -0.00010400

H 0.85859600 -3.82850800 -0.00024400
H -1.69630900 1.04918500 0.00005000
C -3.97139100 -1.12976000 0.00004400
C -4.39830200 -2.43926500 0.00003000
C 3.13370000 -1.65622100 -0.00017400
H 3.82151500 -2.49622300 -0.00022600
C 3.57701100 -0.35062600 -0.00009600
S -3.09716600 -3.56006700 -0.00011800
S 2.26558200 0.79057200 -0.00004300
C 4.95427700 0.11658100 -0.00008300
N 5.14082700 1.43180800 -0.00006800
C -4.89463200 0.00909500 0.00012500
N -6.19501500 -0.27075600 0.00031000
C -6.60321900 2.11988500 -0.00016800
C 7.52337900 0.99231400 0.00027100
N 5.90352300 -0.81594300 -0.00014000
N -4.35206900 1.21864600 0.00006900
N 6.43353500 1.83679300 -0.00003400
N 7.17359600 -0.34251300 -0.00024200
N -5.23127600 2.25201200 -0.00000300
N -7.01930300 0.80339700 0.00057600
O -7.36679000 3.06094800 -0.00048100
O 8.67182900 1.38225600 0.00061400
C -4.62605100 3.56512900 -0.00036100
H -5.41705300 4.31499500 0.00013300
H -3.99842500 3.67866700 0.89080900
H -3.99949700 3.67878100 -0.89228200
C -8.43066500 0.48667600 0.00031900
H -8.99830600 1.41700500 0.00474300
H -8.67248500 -0.10007500 -0.89292100
H -8.67047600 -0.10770700 0.88896300
C 6.62116300 3.27060500 0.00033600
H 7.69013500 3.48314200 -0.00118300
H 6.14799800 3.70039400 -0.88963200
H 6.15077900 3.69979700 0.89209300
C 8.19919900 -1.36205600 0.00007300
H 8.08668900 -1.99220000 -0.88924300
H 9.17509500 -0.87686200 -0.00284500
H 8.09041900 -1.98847700 0.89253300
H -5.42227800 -2.79141600 0.00008000

IIA

C -2.28435700 0.69637800 -0.00014900
C -0.95103000 0.40331200 -0.00006800
C -1.28974500 -2.00997800 0.00002600
C -2.67021400 -1.72295500 -0.00005900
C -3.16220800 -0.42132600 -0.00014400
C 0.45408700 0.92619500 -0.00001900

C -0.45408700 -0.92619500 0.00001900
C 0.95103000 -0.40331200 0.00006800
C 1.28974500 2.00997800 -0.00002600
C 2.67021400 1.72295500 0.00005900
H 3.38506400 2.54176900 0.00005800
C 3.16220800 0.42132600 0.00014400
C 2.28435700 -0.69637800 0.00014900
H -2.68518900 1.70563100 -0.00021500
H -3.38506400 -2.54176900 -0.00005800
H 2.68518900 -1.70563100 0.00021500
C -4.62319600 -0.18980000 -0.00021600
C -7.30871000 0.25003200 -0.00036400
C 4.62319600 0.18980000 0.00021600
C 7.30871000 -0.25003200 0.00036400
N -5.41223000 -1.26041800 -0.00015100
N -6.74225500 -1.00800800 -0.00030800
N -6.37167900 1.26049300 -0.00019400
N -5.02968500 1.07325800 -0.00026900
N 5.02968500 -1.07325800 0.00026900
N 5.41223000 1.26041800 0.00015100
N 6.37167900 -1.26049300 0.00019400
N 6.74225500 1.00800800 0.00030800
O 8.50518400 -0.44481100 0.00022000
O -8.50518400 0.44481100 -0.00022000
C -7.58350000 -2.18489900 -0.00008500
H -7.37054600 -2.78510500 0.89155500
H -8.62669700 -1.86934900 -0.00128600
H -7.36882200 -2.78648300 -0.89036000
C -6.79174500 2.64454100 -0.00026400
H -6.39836700 3.14536900 -0.89196700
H -7.88110600 2.67945900 0.00111300
H -6.39599600 3.14600000 0.89001100
C 6.79174500 -2.64454100 0.00026400
H 6.39599600 -3.14600000 -0.89001100
H 7.88110600 -2.67945900 -0.00111300
H 6.39836700 -3.14536900 0.89196700
C 7.58350000 2.18489900 0.00008500
H 7.36882200 2.78648300 0.89036000
H 8.62669700 1.86934900 0.00128600
H 7.37054600 2.78510500 -0.89155500
H 0.94064600 3.03986600 -0.00009200
H -0.94064600 -3.03986600 0.00009200

IIB

C -1.89880100 -0.06101100 -0.00002800
C -0.75100800 -0.79907600 -0.00003300
C -1.90925100 -2.94418100 -0.00001900

C -3.10337900 -2.19523600 -0.00001500
C -3.11092200 -0.80378300 -0.00001800
C 0.75100800 -0.79907600 -0.00003600
C -0.74845100 -2.21827100 -0.00002800
C 0.74845100 -2.21827100 -0.00003200
C 1.89880100 -0.06101100 -0.00003500
C 3.11092200 -0.80378300 -0.00003000
C 3.10337900 -2.19523600 -0.00002800
C 1.90925100 -2.94418100 -0.00002800
H -1.92363100 1.02455900 -0.00002900
H -4.05844900 -2.71399000 -0.00000700
H 1.93999200 -4.03120600 -0.00002500
C -4.40022900 -0.07817500 -0.00000200
C -6.76475800 1.26896200 0.00006100
C 4.40022900 -0.07817500 -0.00002000
C 6.76475800 1.26896100 0.00003000
N -5.51257200 -0.80722900 0.00004500
N -6.67162300 -0.10750100 -0.00001200
N -5.53454500 1.88996100 0.00002600
N -4.34162200 1.24733200 -0.00006100
N 5.51257200 -0.80722900 0.00004400
N 4.34162200 1.24733200 -0.00006100
N 6.67162300 -0.10750100 -0.00001900
N 5.53454500 1.88996100 0.00003100
O 7.81862200 1.86805700 0.00012000
O -7.81862200 1.86805700 0.00000100
C -7.86981400 -0.91757500 0.00006600
H -7.87905900 -1.55477800 0.89143600
H -8.73803200 -0.25871900 -0.00075200
H -7.87822800 -1.55599700 -0.89042600
C -5.44710100 3.33340300 -0.00012200
H -4.90360200 3.66618100 -0.89152200
H -6.45633100 3.74503500 0.00065200
H -4.90218200 3.66617800 0.89039800
C 7.86981400 -0.91757500 0.00015800
H 7.87817400 -1.55616000 -0.89021500
H 8.73803200 -0.25871900 -0.00082600
H 7.87911200 -1.55461500 0.89164600
C 5.44710100 3.33340200 -0.00002100
H 4.90225500 3.66613100 0.89056200
H 6.45633200 3.74503400 0.00069400
H 4.90352900 3.66622800 -0.89135800
H 1.92363100 1.02455900 -0.00003500
H -1.93999200 -4.03120600 -0.00001500
H 4.05844900 -2.71399000 -0.00002500

IIIA

C 2.52710100 -1.11467700 -0.02090300

C 1.19975300 -0.70625400 -0.02017900
C 1.19126300 0.74205200 -0.02029100
C 2.55807500 1.17203800 -0.02108600
C 3.36339100 0.04838400 -0.02126900
C -0.00772800 -1.44203900 -0.01972900
C 0.00761900 1.44170100 -0.01985500
C -1.19987900 0.70591600 -0.01929600
C -1.19137500 -0.74239000 -0.01929400
C -2.55817500 -1.17239500 -0.01894700
H -2.90814800 -2.19983300 -0.01874800
C -3.36349400 -0.04874500 -0.01854400
C -2.52722100 1.11434300 -0.01896400
H 0.00879000 -2.53325900 -0.01973800
H 2.89456500 -2.13694000 -0.02094500
H 2.90810300 2.19945700 -0.02129600
H -0.00890200 2.53292100 -0.01994200
H -2.89478400 2.13657600 -0.01877800
C 4.82121300 0.02656100 -0.02110300
C 7.53040900 -0.02333200 0.02279700
C -4.82128600 -0.02682200 -0.01682200
C -7.53126400 0.02353700 0.02242500
N 5.44173000 1.20651300 -0.02016500
N 6.79497100 1.14053800 -0.02327900
N 6.75719900 -1.16032700 -0.02597600
N 5.39977700 -1.16883100 -0.02223900
N -5.44188000 -1.20691600 -0.01493600
N -5.40007300 1.16818500 -0.01737400
N -6.79477700 -1.14009100 -0.01675100
N -6.75762200 1.16066700 -0.01695500
O -8.74298000 0.02553000 0.07616600
O 8.74153500 -0.02947300 0.08754800
C 7.50212800 2.40153400 0.03200600
H 7.90069200 2.58172700 1.03853500
H 8.33897400 2.38780100 -0.67196800
H 6.78870700 3.18408200 -0.23021700
C 7.38629400 -2.46024500 0.03514900
H 6.83218300 -3.13944300 -0.61804300
H 8.42252900 -2.36790100 -0.29325500
H 7.36460200 -2.85254900 1.06037600
C -7.50477400 -2.40004200 0.02859100
H -7.95024300 -2.56029800 1.01840100
H -8.30865700 -2.40126800 -0.71348400
H -6.77997200 -3.18645200 -0.18600900
C -7.38095400 2.46368100 0.03180800
H -6.89766100 3.10984700 -0.70691600
H -8.44293000 2.35368200 -0.19051300
H -7.25815500 2.90838800 1.02759800

IIIB

C -1.61511100 0.37363100 0.00001700
C -0.47246500 -0.48247400 0.00001200
C -0.95643000 -1.83752500 -0.00013900
C -2.35962700 -1.78434300 -0.00022700
C -2.75774500 -0.42270100 -0.00009900
C 0.88279200 -0.19870500 0.00008400
C -0.05792900 -2.91470400 -0.00019600
C 1.29653300 -2.63313200 -0.00013400
C 1.78387200 -1.27823300 -0.00000300
C 3.20209600 -1.35621400 0.00005900
C 3.57722900 -2.73125900 -0.00001200
C 2.43525800 -3.50110300 -0.00015100
H 1.24643100 0.82658500 0.00018800
H -1.60665900 1.45936200 0.00011900
H -3.04241900 -2.62912300 -0.00034400
H -0.41736200 -3.94504700 -0.00030300
H 2.39541700 -4.58654900 -0.00025300
C -4.13532900 0.05104300 -0.00013700
C -6.70735200 0.93001700 0.00023800
C 4.16666300 -0.27063700 0.00014700
C 6.01273000 1.72740200 -0.00014100
N -5.08823900 -0.87557200 -0.00035000
N -6.35919900 -0.40389400 -0.00052200
N -5.61528700 1.77237300 -0.00011500
N -4.32128700 1.36901600 -0.00001900
N 3.70565700 0.98070100 0.00000600
N 5.45152800 -0.62910800 0.00035500
N 4.65070000 1.95003700 -0.00010900
N 6.34256500 0.38679900 0.00060900
O 6.83441200 2.61698800 -0.00067300
O -7.85521300 1.32266000 0.00099400
C -7.38482400 -1.42294800 -0.00002000
H -7.27753500 -2.04873300 0.89314300
H -8.36070100 -0.93762100 -0.00430800
H -7.27207000 -2.05423400 -0.88854500
C -5.80359900 3.20592800 0.00072700
H -5.32895800 3.63634800 -0.88819000
H -6.87244600 3.41909000 -0.00246600
H -5.33481100 3.63499800 0.89345500
C 4.13512200 3.30179500 -0.00063400
H 3.51667300 3.45634200 0.89058500
H 4.97437600 3.99712900 -0.00026600
H 3.51768000 3.45613000 -0.89259800
C 7.73128400 -0.02082900 0.00023700
H 7.93398800 -0.62159500 -0.89320800
H 8.35817800 0.87064400 0.00474600
H 7.93155600 -0.62930100 0.88892200
H 4.60100400 -3.08412100 0.00001600

IVA

C 6.04084000 -0.00008500 0.00024000
C 8.75731400 0.00008600 -0.00002900
C -6.04083900 0.00008300 -0.00072000
C -8.75730800 -0.00008100 0.00067100
N 6.64173900 1.18551900 0.00027700
N 7.99646700 1.15051200 0.00044600
N 7.99660800 -1.15044400 0.00029200
N 6.64189100 -1.18563100 0.00028100
N -6.64188600 1.18563200 -0.00097500
N -6.64174000 -1.18552000 -0.00074600
N -7.99660300 1.15044700 -0.00133400
N -7.99646800 -1.15050900 -0.00096800
O -9.96990000 -0.00014500 0.00262800
O 9.96990700 0.00015700 -0.00051400
C 8.63736600 2.44708900 -0.00007700
H 8.32693400 3.00730800 0.88898800
H 9.71778200 2.30375600 0.00315200
H 8.33213600 3.00452700 -0.89274700
C 8.63768000 -2.44693600 -0.00006200
H 8.33084500 -3.00521200 -0.89163800
H 9.71808000 -2.30345700 0.00109900
H 8.32899200 -3.00640100 0.89010400
C -8.63768800 2.44693100 0.00058900
H -8.31967700 3.01116700 -0.88315000
H -9.71802100 2.30350000 -0.01215300
H -8.34025400 3.00038300 0.89844700
C -8.63737800 -2.44707900 0.00087900
H -8.33685700 -3.00177200 0.89690800
H -9.71776400 -2.30376600 -0.00818800
H -8.32227700 -3.01002000 -0.88476000
C 2.42228700 -0.72635900 0.00002900
C 1.22452100 -1.41829700 -0.00011100
C -0.00001700 -0.72439900 -0.00014000
C 0.00001800 0.72439500 -0.00001900
C 1.22445400 1.41820600 0.00011500
C 2.42231500 0.72619400 0.00013600
C -1.22445300 -1.41821000 -0.00030900
C -1.22452000 1.41829300 -0.00006600
C -2.42228600 0.72635500 -0.00025100
C -2.42231400 -0.72619800 -0.00037600
C -3.76830800 -1.14709500 -0.00057700
C -4.58027700 0.00016400 -0.00055800
C -3.76841700 1.14729800 -0.00036400
H -1.21080600 -2.50977900 -0.00040500
H 1.21078300 -2.50986500 -0.00020000
H 1.21080700 2.50977500 0.00019800
H -1.21078200 2.50986000 0.00003200

H -4.13023900 -2.16995000 -0.00070600
H -4.13028200 2.17017400 -0.00031300
C 3.76830900 1.14709200 0.00024300
C 3.76841800 -1.14730200 0.00008900
C 4.58027800 -0.00016800 0.00019200
H 4.13028400 -2.17017700 0.00003800
H 4.13024000 2.16994600 0.00033000

IVB

C 5.36333700 0.21704300 -0.00030800
C 7.98511900 0.92725500 0.00000800
C -5.30198100 -0.11189000 0.00001600
C -6.97475300 2.03628200 -0.00032500
N 6.25331700 -0.77027100 -0.00061900
N 7.55182700 -0.38206700 -0.00142000
N 6.95000300 1.83893400 -0.00000800
N 5.63316800 1.51898700 -0.00021700
N -6.61547400 -0.36050300 -0.00002800
N -4.73850300 1.09782400 -0.00009300
N -7.41626500 0.72925000 -0.00024900
N -5.59930900 2.14307600 -0.00024100
O -7.71856800 2.99301700 -0.00042800
O 9.15548300 1.24452000 0.00104200
C 8.50951200 -1.46551900 -0.00051200
H 8.35105700 -2.09208500 -0.88530600
H 9.51481100 -1.04464900 -0.01185400
H 8.36655900 -2.07895600 0.89624600
C 7.23018900 3.25760000 0.00082600
H 6.78698600 3.71642400 0.89178300
H 8.31053900 3.40146200 0.00119500
H 6.78747400 3.71734200 -0.88990200
C -8.83419000 0.44057400 -0.00023400
H -9.08644000 -0.14450000 0.89108100
H -9.38372000 1.38170200 -0.00107100
H -9.08607200 -0.14592600 -0.89070400
C -4.97210200 3.44632500 -0.00034800
H -4.34329600 3.54881300 -0.89196800
H -5.74986500 4.20983000 -0.00037500
H -4.34324100 3.54892300 0.89121800
C 1.67723400 -0.03591500 0.00011800
C 0.34455600 0.31856100 0.00016400
C -0.65801200 -0.67664900 0.00023200
C -0.27791800 -2.06690200 0.00029200
C 1.08729200 -2.41721700 0.00023500
C 2.05816000 -1.43415000 0.00012800
C -2.02184600 -0.31921600 0.00020400
C -1.28600200 -3.05427000 0.00036700
C -2.61474300 -2.69194500 0.00035000
C -2.99907800 -1.29776700 0.00024200

C -4.43075000 -1.26637800 0.00019000
C -4.89273900 -2.60523500 0.00029500
C -3.81138100 -3.46811100 0.00038800
H -2.29277400 0.73451100 0.00014000
H 0.04305300 1.36730300 0.00013000
H 1.36099700 -3.47359700 0.00025900
H -0.99498600 -4.10592700 0.00042400
H -3.85884200 -4.55260100 0.00047300
C 3.47301600 -1.48567700 -0.00002300
C 2.87315500 0.72768500 0.00000300
C 3.95511700 -0.16473400 -0.00009600
H 2.95266000 1.80963300 -0.00003000
H 4.09101800 -2.37737800 -0.00007800
H -5.93973700 -2.88571100 0.00028600

VA

C 5.52283600 0.12081100 -0.00018300
C 8.21437900 -0.24012900 0.00019500
C -5.52283600 -0.12081300 0.00011100
C -8.21437900 0.24013300 0.00055600
N 6.27602800 1.21364400 -0.00021300
N 7.61364700 1.00115800 -0.00025200
N 7.30733500 -1.28124900 -0.00002300
N 5.96098600 -1.13910000 -0.00013700
N -5.96098300 1.13910000 0.00015800
N -6.27603100 -1.21364400 0.00002600
N -7.30733100 1.28125200 0.00029800
N -7.61365000 -1.00115400 0.00010000
O -9.41549900 0.40188000 0.00030200
O 9.41550000 -0.40187200 0.00062100
C 8.42075300 2.20162800 0.00010700
H 8.19192000 2.79494700 0.89244600
H 9.47254300 1.91605300 -0.00226700
H 8.18837800 2.79744200 -0.88960500
C 7.77307500 -2.65104300 0.00029300
H 7.39343500 -3.16439100 -0.89010600
H 8.86289600 -2.65092200 -0.00038000
H 7.39459800 -3.16371400 0.89159000
C -7.77306900 2.65104700 0.00021600
H -7.39401400 3.16400100 -0.89066400
H -8.86289000 2.65092800 0.00023100
H -7.39400500 3.16411000 0.89103200
C -8.42075700 -2.20162300 -0.00007600
H -8.19084600 -2.79592600 0.89131600
H -9.47254900 -1.91604400 -0.00093000
H -8.18946200 -2.79645600 -0.89074000
C 2.08525200 1.45038700 -0.00020800

C 1.83247000 0.01979000 -0.00020800
C 0.55259700 -0.48466300 -0.00018900
C -0.55259700 0.48465800 -0.00015200
C -0.27483300 1.89912000 -0.00015400
C 0.99468700 2.37150300 -0.00018500
C 0.27483200 -1.89912400 -0.00019900
C -1.83247000 -0.01979500 -0.00011400
C -2.08525200 -1.45039100 -0.00012600
C -0.99468800 -2.37150700 -0.00017000
C -3.44202400 -1.63578300 -0.00008200
C -4.08159500 -0.32436000 -0.00000400
C -3.11987300 0.64571300 -0.00005800
H 1.11996700 -2.58277900 -0.00023300
H -1.11996800 2.58277400 -0.00013300
H 1.19228700 3.44259500 -0.00018800
H -1.19228800 -3.44259900 -0.00018100
H -3.97897000 -2.57890100 -0.00007200
H -3.30116100 1.71376000 -0.00003000
C 3.44202300 1.63578000 -0.00022600
C 4.08159400 0.32435600 -0.00021600
C 3.11987300 -0.64571700 -0.00021700
H 3.30116000 -1.71376400 -0.00020800
H 3.97897000 2.57889700 -0.00022800

VB

C 5.13932600 0.17710900 0.02741200
C 7.58028300 1.34987800 -0.00800600
C -5.09732200 -0.02751300 -0.02103600
C -7.21744000 1.66778500 0.00762400
N 6.19335900 -0.63144400 0.03594300
N 7.39890100 -0.01428100 0.04858100
N 6.40283100 2.06121300 0.03956600
N 5.16333600 1.51066200 0.02641900
N -6.31648900 -0.57860800 -0.02251600
N -4.82877200 1.28311400 -0.02319200
N -7.34565500 0.29684100 -0.02653100
N -5.90848800 2.09929000 -0.03194800
O -8.17240300 2.41073900 0.05715100
O 8.67135600 1.87411800 -0.07864500
C 8.55627700 -0.87944800 -0.01264700
H 8.86267500 -1.05088300 -1.05307700
H 9.38481400 -0.41315900 0.52475500
H 8.28180200 -1.83114500 0.44658300
C 6.45770300 3.50492400 -0.02984500
H 5.52031100 3.88837400 0.37712600
H 7.31019400 3.86481600 0.55087900
H 6.57269500 3.84316900 -1.06808600
C -8.67871200 -0.26729700 0.01595700

H -9.13959800 -0.09984300 0.99707200
H -9.30769700 0.20433700 -0.74495400
H -8.58123000 -1.33690200 -0.17396700
C -5.61488300 3.51498500 0.01726100
H -4.79155300 3.72008500 -0.67247200
H -6.50703000 4.07112300 -0.27319700
H -5.31512600 3.80929600 1.03116500
C 2.25432000 -2.13530200 0.00995000
C 1.58775400 -0.86742600 0.00724400
C 0.20415600 -0.77547500 0.00070400
C -0.54817400 -2.02932200 -0.00360000
C 0.14531000 -3.28251800 -0.00117400
C 1.50618800 -3.34108100 0.00534300
C -0.49536700 0.47075100 -0.00167500
C -1.93146200 -1.93682400 -0.00964000
C -2.60627500 -0.67754000 -0.01183100
C -1.85719100 0.52992400 -0.00771700
C -3.98806600 -0.95076900 -0.01730300
C -4.16590400 -2.39334500 -0.01835800
C -2.94675600 -2.98590600 -0.01404500
H 0.08957600 1.38742500 0.00167000
H -0.44282100 -4.19664700 -0.00465700
H 2.02111800 -4.30086800 0.00720700
H -2.37205200 1.48559200 -0.00904500
H -2.75446900 -4.05298700 -0.01392000
C 3.62398200 -1.89338500 0.01760100
C 3.83268800 -0.46464800 0.01965100
C 2.61298900 0.16250200 0.01344300
H 2.45663400 1.23515400 0.01356000
H 4.41927800 -2.63225000 0.02142500
H -5.13282800 -2.88140700 -0.02224200

VIA

C 5.88441500 0.00000000 -0.00007300
N 6.49752800 1.18260300 -0.00085300
C -5.88435600 0.00000000 -0.00021900
N -6.49759900 1.18262500 0.00075900
C -8.61272200 0.00000000 0.00013100
C 8.61270800 0.00000100 0.00025900
N 6.49752900 -1.18260400 0.00047100
N -6.49759900 -1.18262300 -0.00125900
N 7.85014000 1.14793600 -0.00082700
N 7.85014100 -1.14793500 0.00004900
N -7.85016200 -1.14792000 -0.00026700
N -7.85016100 1.14791900 -0.00041700
O -9.82583700 0.00000700 0.00074000
O 9.82580400 0.00000000 0.00092100

C -8.48935400 -2.44551100 -0.00143500
H -9.56994200 -2.30347400 0.00670500
H -8.18741900 -3.00041800 -0.89685100
H -8.17436400 -3.00737200 0.88496300
C -8.48936600 2.44550300 0.00188100
H -8.16942800 3.01007400 -0.88095500
H -9.56989600 2.30350800 -0.01244100
H -8.19244700 2.99765300 0.90073200
C 8.48929200 2.44551100 -0.00103600
H 8.18250000 3.00342900 0.89080500
H 9.56990200 2.30343200 -0.00302000
H 8.17918300 3.00441600 -0.89107000
C 8.48929700 -2.44550700 0.00157800
H 9.56989200 -2.30344200 -0.00491100
H 8.18603900 -3.00079400 0.89630500
H 8.17567500 -3.00702700 -0.88553300
C -4.40889300 0.00000100 -0.00016000
C -3.75039700 -1.24066900 -0.00012700
C -3.75039600 1.24066900 -0.00013100
C -2.40692800 -1.53866600 -0.00005600
H -4.40817000 -2.10846800 -0.00012400
C -2.40692600 1.53866600 -0.00012000
H -4.40816900 2.10846800 -0.00016300
C -1.24211600 -0.72145200 -0.00003300
H -2.18279500 -2.60648500 -0.00000500
C -1.24211600 0.72145100 -0.00010700
H -2.18279300 2.60648500 -0.00014500
C 4.40870400 -0.00000100 -0.00006400
C 3.75048400 -1.24072300 0.00015500
C 3.75048300 1.24072200 -0.00027300
C 2.40694900 -1.53875000 0.00018100
H 4.40829800 -2.10845700 0.00030400
C 2.40695000 1.53874900 -0.00028900
H 4.40829800 2.10845600 -0.00042600
C 1.24222900 -0.72147600 0.00004100
H 2.18282300 -2.60656400 0.00034100
C 1.24222800 0.72147500 -0.00014000
H 2.18282400 2.60656400 -0.00044800
C 0.00003500 -1.36304200 0.00007200
C 0.00003600 1.36304200 -0.00017500
H 0.00004300 -2.45240100 0.00017000
H 0.00004400 2.45240000 -0.00027200

VIB

C -5.38850000 0.02444100 -0.00018900
N -5.14232400 1.32904400 -0.00119800
C 5.44086000 0.23693000 -0.00015500
N 5.74285500 1.53653500 0.00028200

C 8.08568400 0.91532200 0.00060600
C -7.53815400 1.69351000 0.00012500
N -6.59047400 -0.53655300 0.00080000
N 6.33136900 -0.75707400 -0.00054100
N -6.23109300 2.13437100 -0.00176400
N -7.64120300 0.31914700 0.00131300
N 7.63276900 -0.38597800 -0.00065000
N 7.06227100 1.83775400 0.00047500
O 9.26097100 1.21679600 0.00154100
O -8.49447200 2.43726300 0.00061700
C 8.57476300 -1.48325400 -0.00035000
H 9.58602900 -1.07675700 -0.00542300
H 8.41882400 -2.09819400 0.89316200
H 8.41171100 -2.10380700 -0.88859900
C 7.35990300 3.25293400 0.00168900
H 6.92505900 3.71673500 0.89421500
H 8.44201300 3.38311000 -0.00063500
H 6.92078400 3.71878400 -0.88762400
C -5.93930600 3.55069400 -0.00235200
H -5.34778100 3.80098500 -0.89015100
H -6.87975600 4.10156200 -0.00945700
H -5.35940600 3.80513100 0.89204600
C -8.94061900 -0.31514500 0.00265100
H -9.70778700 0.45907400 0.00570900
H -9.04158500 -0.94349100 -0.88945600
H -9.03766500 -0.94692400 0.89271400
C 4.01702700 -0.12814000 -0.00023000
C 3.68542700 -1.49504700 -0.00043500
C 3.06742100 0.91339600 -0.00009000
C 2.45903400 -2.11627200 -0.00048400
H 4.53786500 -2.17247700 -0.00054900
C 1.69571000 0.86926400 -0.00013700
H 3.49024900 1.91671300 0.00005600
C 1.12657900 -1.60986100 -0.00037100
H 2.50510200 -3.20627100 -0.00062600
C 0.76795600 -0.21687600 -0.00026100
H 1.21189100 1.84712700 -0.00003400
C -4.50144400 -2.26919100 -0.00002900
C -3.58178000 -3.31823500 -0.00010100
C -4.21285100 -0.89737500 -0.00018100
C -2.20431800 -3.30936900 -0.00024100
H -4.02819900 -4.31432000 -0.00003500
C -2.96173100 -0.29564100 -0.00031300
C -1.28081600 -2.22588500 -0.00030000
H -1.73166200 -4.29239400 -0.00027200
C -1.64395100 -0.83023800 -0.00028200
H -2.98478700 0.79148000 -0.00041900
C 0.08092700 -2.53897100 -0.00037700
C -0.59279700 0.09721700 -0.00025600
H 0.35020000 -3.59470900 -0.00043000

H -0.86104200 1.15300800 -0.00020900
H -5.55138600 -2.54280000 0.00010900
